# Supplementary material for: Hierarchical lifelong topic modeling using rules extracted from network communities
Source: PLoS One. 2022 Mar 3;17(3):e0264481. doi: 10.1371/journal.pone.0264481 (PMC8893656; doi:10.1371/journal.pone.0264481)

Manuscript Click here to access/download;Manuscript;CNLMLjournal.pdf

Hierarchical Lifelong Topic Modeling Using Rules Extracted From Network Communities

Muhammad Taimoor Khan[a], Nouman Azam[a], Furqan Aziz[b] and Shehzad Khalid[c]

[a]*Department of Computer Science, National University of Computer and Emerging Sciences, Peshawar 25000, Pakistan*
[b]*Department of Computer Science, IMSciences, Peshawar, 25000, Pakistan*
[c]*Department of Computer Engineering, Bahria University, Islamabad, 44000, Pakistan*

Abstract

Topic Models extract latent concepts from texts in the form of topics. Lifelong topic models extends topic models by learning topics continuously based on accumulated knowledge from the past which is updated continuously as new information becomes available. On the other hand, hierarchical topic modeling extends topic modeling by extracting topics and organizing them into a hierarchical structure. In this study, we combine the two and introduce hierarchical lifelong topic models. The hierarchical lifelong topic models not only allow to examine the topics at different levels of granularity but will continuously adjust the granularity of the topics as more information becomes available. A fundamental issue in hierarchical lifelong topic modeling is the extraction of rules that will preserve the hierarchical structural information among the rules and will continuously update it as new information is available. To address this issue, we introduce a network communities based rule mining approach for hierarchical lifelong topic models or NHLTM. The proposed approach extracts hierarchical structural information among the rules by representing textual documents as graphs and analyzing the underlying communities in the graph. An architecture of hierarchical lifelong topic modeling is also introduced which realizes the proposed approach for extracting rules. Experimental results indicate plausibility of the hierarchical topic structures for maintaining high topic coherence that increases from -856 for general topics to -804 for specific topics.

these are not meaningful #s

*Corresponding Author: Email: taimoor.khan@nu.edu.pk (M. Taimoor Khan);

Keywords: Topic modeling, lifelong learning, lifelong topic modeling, community detection

---

1. Introduction

Topic models process a collection of documents to extract hidden thematic structures, called topics [1, 2]. Each topic is an ordered lists of contextually correlated words representing a concept. Topic modeling has many extensions based on the learning paradigm such as supervised, semi-supervised, transfer learning, hybrid and knowledge-based models. These extensions are designed to meet the specific demands and needs found in different application areas [3, 4, 5]. In this paper we examine a new type of topic models called hierarchical lifelong topic models.

Lifelong topic models (LTM) is an important extension to topic models where topics are continuously learned and refined based on previously accumulated knowledge and processing of new information [3, 6]. Unlike other extensions, it does not require external guidance to analyze a dataset or to mine rules from it. More specifically, it exploits the huge volume of data processed in the past and the overlap in their concepts to identify global patterns and mine them as rules [7]. Such an approach is highly desired in unsupervised analysis of continuously arriving data from diverse sources [8]. Hierarchical topic modeling is another exciting extension which organizes the topics in a hierarchical structure, thereby allowing to view the topics at different levels of granularity. The topics at higher levels are general and abstract and get more specific down the hierarchy [9]. The incorporation of hierarchical topics in lifelong topic models will allow for examining the topics at different levels of granularity and adjusting the granularity as new information becomes available. This may be useful in applications such as summarization of news stories at different levels based on continuous streaming of news items.

A fundamental issue for incorporating hierarchical topics in lifelong long topic models is the efficient and effective extraction of rules that are used to construct topics. Existing approaches are not suitable for this task. On one hand, the rule extraction approaches in LTM models facilitate in producing coherent topics but are not well suited to arrange the topics into a hierarchy [8, 10, 11, 12]. In particular, they provide little or no information regarding the structural relationship that may occur among the topics [13, 14]. On

the other hand, the rule extraction approaches in hierarchical topic models do not benefit from the past experience and rather rely on some level of supervision or manual guidance. Moreover, such support is required each time, when new information is processed in the form of new dataset. To overcome these issues, we present an approach called network communities based rule mining for hierarchical lifelong topic models or NHLTM.

The proposed NHLTM approach extract rules that provides the hierarchical structure among the rules and update the hierarchical relationships among the rules as more information becomes available. In particular, rules are extracted by constructing a graph based on the data in textual documents and then communities of related words are detected within the graph. The edges within a community helps in identifying useful and important rules while the edges between communities help in identifying the hierarchical association among the rules. The approach continuously updates the communities based on new information which result in updated hierarchical information among the rules. Experimental results on Chen2014 dataset suggests a higher relevance to human judgment for easily interpretable topics [15]. The hierarchical structure of the topics is rationalized by the gradual improvement of topic coherence across the topics at each level. The proposed approach has topic coherence improved that is from -856 to -804 down the hierarchy. The empirical log-likelihood of the held-out test documents flattens at -113, indicating stable behavior of the model for predicting the unseen data at varying levels and number of topics in the hierarchy.

2. Topic Models, Lifelong and Hierarchical Topic Models

2.1. Topic Models

A topic consist of contextually related words that collectively represents a concept [16, 17]. An inference technique is typically used that iteratively determines the distribution of words across different topics based on words relationships in documents [18]. Words that co-occur across many documents have high chance of being selected under the same topic [19]. Topic models are used in different applications to explore, categorize, summarize and analyze textual data [20].

Topic models have been extended in variety of ways and lead to models such as supervised models, hybrid models, transfer learning models, semi-supervised models, knowledge-based models and lifelong learning models [17, 21, 22]. In supervised models, the documents in training data are tagged

with manually provided set of topic labels [23, 24]. Semi-supervised topic models incorporate expert guidance in associating certain words to topics while the other words are grouped around them in their respective topics [7]. Hybrid topic models are trained on certain features using a small labeled data and is provided for unsupervised analysis on remaining data. It is used in case of limited availability of labeled training data. The supervised and hybrid topic models help to produce interpretable topics, however, its use is only limited to the datasets for which such labeled training data exists. Transfer learning based topic models are trained on one dataset and applied on another having limited or noisy documents [25, 26]. Knowledge-based topic models are manually provided with set of rules that constraint the model to observe them while extracting topics [10, 27, 28]. The inference technique i.e., Gibbs sampling is modified in knowledge-based topic models where it has to incorporate the rules impact as well, as external guidance, in deciding the associations among words for a topic. Lifelong learning approach with topic modeling can be considered as the knowledge-based topic modeling where the rule mining mechanism is automated [29].

Hierarchical topic modeling uses some level of supervision or external guidance to combine the topics of a dataset into hierarchical structure [30]. The high level topics that are closer to root are general topics while the topics away from root are specialized topics. The literature of lifelong topic modeling and hierarchical topic modeling is presented below and their shortcomings are highlighted.

2.2. Lifelong Topic Modeling

Lifelong topic models continue to mine new rules from the dataset of each task, while it benefits from the relevant rules that were mined in the previous tasks [31, 29]. Considering the diversity of the datasets processed in the past, only the rules that are relevant to the current task can be of help [6, 32, 14, 3]. For this purpose, the background or context of a rule is compared with the vocabulary of the current dataset. It may be considered as resolving polysemy at the rule level. Although the quality of automatically mined rules may not be as good as that of a human expert, however, it is compensated by the quantity of rules and the freedom that it can be applied to data of any nature [33, 34, 11].

Generally speaking, the lifelong learning cycle contains four main components [35]. The first component deals with mining rules from a task completed. The rules are first evaluated based on some evaluation criteria reflect-

ing their quality. Important rules are mined based on suitable thresholds on the evaluation criteria. The rules may represent semantic or contextual correlations among words [28, 36]. The approaches used for evaluating the quality of rules in literature are Normalized point-wise mutual information, frequent itemset mining, multi-support frequent itemset mining etc [11, 12, 8]. This components executes after performing each task of extracting topics from a dataset.

The second component deals with representing the rules in a format where they can be efficiently and effectively retrieved when required [28]. In literature, the rules are represented as word pairs with positive correlation and strength of correlation, called must-link rules. For example, the must-link rule $mustLink(word_1, word_2, +, 0.67)$ indicates a positive correlation between the words $word_1$ and $word_2$, indicated by the + sign. This rule adds bias into the gibbs sampling based inference to increase the probability of $word_2$ for topics where $word_1$ has higher probability and vice versa with an impact of 0.67 [29]. Similarly, the rules with word pairs having negative correlation with a strength of negative correlation is called cannot-link rules.For example, the cannot-link rule $cannotLink(word_1, word_3, -, 0.86)$ indicates a negative correlation between the words $word_1$ and $word_3$, shown by the $-$ sign. The rule adds a bias into the gibbs sampling based inference to decrease the probability of $word_3$ for topics where $word_1$ has high probability and vice versa with an impact of 0.86 [31, 11, 8]. Thus, in the modified gibbs sampling the intuition of arranging words into topics comes from the current dataset and relevant rules of the past [31].

The third component deals with the retention and maintenance of rules to preserve them for future [29]. The rules are maintained with different levels of abstraction, providing a tradeoff between storage space and efficiency [31, 33, 8, 11]. Lifelong learning can also be considered as a longer chain of transfer learning with unknown target datasets, multiple source datasets and that can be processed in any sequence. Therefore, it demands perseverance of rules for longer duration. For example, the rules learnt from 2nd task may be utilized by the 10th or 50th task. On the other hand, the rules mined automatically are expected to have wrong, irrelevant, duplicate and even contradictory rules. Therefore, the retention and maintenance module undergo a refining process to resolve these issues and keep only consistent and good quality rules.

The fourth component deals with the transfer of rules relevant to the current task and benefiting from it by incorporating the bias from the rules [10]. The gibbs sampling inference technique is extended with the Generalized

polya urn (GPU) model [11]. The GPU model and its other variants are responsible for introducing the bias for different types of rules into gibbs sampling [31, 29, 33, 37, 27].

2.3. Hierarchical Topic Modeling

Hierarchies are highly important in displaying the various components of a system as a downwards tree that has generic concepts at higher levels while specific concepts at lower levels [30]. Topics that are structured in a hierarchy can be analyzed at different levels of granularity and compared to one another [38]. There are some studies on hierarchical topic modeling with traditional topic modeling approaches [30, 9, 25]. We refer the approaches that are not using lifelong learning approach as the traditional approaches. They perform hierarchical topic modeling for a dataset in isolation.

The hLDA model is the earliest attempt in organizing topics into a hierarchical structure [39, 13]. It make use of the nested chinese restaurant process (nCRP) to set a prior on possible hierarchical trees using the topic prior β. The gibbs sampling technique is also modified to predict new topic for the sampled word in the given document while it also predict the level of topics associated with the same document. The SSHLDA model organize the topics of a dataset into a hierarchical structure using labeled training data [9]. Transfer learning based topic models are used to transfer the hierarchical structure of one dataset into another related dataset [25].

The notable attempts for extracting topic hierarchies in literature either require external support or hyper-parameters for the hierarchical structure which limits their application to certain scenarios only. Moreover, they perform hierarchical topic modeling in isolation and would require the same support each time. On the other hand, lifelong topic models benefit from the topic modeling performed on the previous datasets to improve the coherence of topics for the relevant future datasets. But the existing lifelong topic modeling approaches make use of statistical measures to mine rules. These approaches mine, maintain and transfer the rules in isolation, which makes them inconclusive towards generating topic hierarchies [11, 12, 8]. Therefore the existing lifelong topic modeling approaches are not well suited for finding the associations among topics [40, 41]. In particular, they lack in conveying structural information which is a key constituent for extracting topic hierarchies [41, 42]. In the next section, we demonstrate this limitation of the existing rule mining approaches and indicate towards a possible solution.

Table 1: Sample data of documents and words

	RAM	*Cache*	*Intel*	*Core*	*Price*	*Grapahics*	*Video*	*keyboard*	*Cost*
D_1	-	-	-	-					
D_2	-	-	-	-		-	-	-	
D_3			-	-	-				-
D_4		-	-	-	-			-	-
D_5	-	-	-	-		-	-		
D_6	-	-				-	-		
D_7	-		-	-	-	-	-	-	-
D_8	-	-			-				-
D_9	-	-			-			-	-
D_{10}						-	-	-	

3. Limitations in Existing Rule Mining Approaches for Topic Modeling

In this section, we highlight a limitation in rule mining approaches that are used in existing lifelong topic modeling approaches. Consider Table 1 which represents a sample dataset. The rows of the table correspond to documents and the columns correspond to words. A "-" in a cell means that the corresponding word appears in the respective document. For instance, *RAM* is present in document D_1, D_2, D_5, D_6, D_7, D_8 and D_9. We are interested in co-occurrences of words across the documents. Table 2 is constructed for this purpose based on data in Table 1. The rows and columns of Table 2 correspond to the words. A particular entry in the table reflects the co-occurrence between a pair of words. For instance, a value of 6 in the second column corresponding to the first row meaning that the words *RAM* and *Cache* has co-occurred in 6 documents. This can be easily seen in Table 1, i.e., *RAM* and *Cache* both occurred together in Documents D_1, D_2, D_5, D_6, D_8 and D_9.

The typical lifelong topic modeling approaches consider rules in the form of word pairs. Moreover, only those rules are considered as rules whose evaluation is above certain threshold value. Lets assume that the words co-occurrence is used as an evaluation measure with a threshold being set to 5. This will result in selection of four rules, namely, $r_1(RAM, Cache)$, $r_2(Intel, Core)$, $r_3(Price, Cost)$ and $r_4(Graphics, Video)$. Since these rules have a co-occurrence frequency of 5 or more. We may note that the rule are evaluated in isolation, irrespective of their associations with other rules. More specifically, it ignores the relationships the words of different rules or simply

Table 2: Words co-occurrence based on Table 1

	RAM	*Cache*	*Intel*	*Core*	*Price*	*Graphics*	*Video*	*Keyboard*	*Cost*
RAM	7	6	4	4	3	4	4	3	3
Cache		7	4	4	2	3	3	3	3
Intel			6	6	3	3	3	3	3
Core				6	3	3	3	3	3
Price					5	1	1	2	5
Graphics						5	5	3	1
Video							5	3	1
Keyboard								5	3
Cost									5

Table 3: Association between different rules according to Equation 1

	r_1	r_2	r_3	r_4
r_1		4	3	3.5
r_2			3	3
r_3				1
r_4				

among different rules. For example, the rules r_1 and r_2 have words having very strong association such as *RAM* and *Intel* has a co-occurrence of 4. This relationship is however not being considered in the existing approaches.

The issue of mining rules in isolation can be addressed by considering the association among the rules in the form of co-occurrence of the words belonging to different rules. We define the associations based on co-occurrence information between words belonging to different rules as,

$$Association(r_i, r_j) = \frac{\sum_{w \in r_i, w' \in r_j} CF(w, w')}{|r_i| \times |r_j|}. \qquad (1)$$

where w and w' are distinct words belonging to different rules r_i and r_j, respectively. The association between the two rules is the summation of all word co-occurrences across the two rules. For instance, consider the rules r_1 and r_2, the association between these two rules according to Equation (1) is,

$$Association(r_1, r_2) = \frac{CF(RAM, Intel) + CF(RAM, Core) + CF(Cache, Intel) + CF(Cache, Core)}{|RAM, Cache| \times |Intel, Core|} \qquad (2)$$

$$= \frac{4+4+4+4}{2 \times 2} = 4$$

The association among rules are calculated for all the other rules as well and are given in Table 3. These associations can be used to find which rules are more closely related to each other as compared to others. Considering all the rules as leaf nodes of a tree, the rules r_1 and r_2 have the highest association of 4 according to Table 3. They are linked at the next higher level to form a more general rule. We call this the rule $r_{1,2}$, which can also be considered as the parent rule for the two rules r_1 and r_2. Now at the next higher level, there are three rules i.e., $r_{1,2}$, r_3 and r_4. Following the same formula, the association among these rules is evaluated where the rules $r_{1,2}$ and r_4 have the next highest association with an association score of 3.25. The association score of rules $r_{1,2}$ and r_3 is 3 and that of rules r_3 and r_4 is 1, respectively. Thus, the rule $r_{1,2,4}$ is the next level parent rule that has the rules $r_{1,2}$ and r_4 as its direct descendants. Finally, the two available rules i.e., $r_{1,2,4}$ and r_3 having association score of 2.3 are linked to form a single rule as $r_{1,2,3,4}$. This is one way of finding the structural association among rules.

Exploiting the structure structural association among rules, a hierarchical structure may be generated for the topics of a given dataset. For instance, consider topics T_1, T_2, ... T_K. The topic T_1 is containing the words of the rule r_1 and T_2 is containing the words of the rule r_2. Since the rules r_1 and r_2 have the closest association, therefore, topics T_1 and T_2 are merged at the higher level to represent a single topic $T_{1,2}$. The structure between the topics can be successively generated by combining the topics containing rules with highest associations. This structure will lead to hierarchy with the dataset as root node and the specific topics as leaf nodes while the intermediate nodes representing general topics at different levels. The topics that do not contain any rule are directly linked to the root of the hierarchy as an outlier.

A critical issue in the above intuitive solution is how to determine and model the association of rules based on sound theoretical notions. The exploration of this issue will open up new ways for exploiting the relationships among the rules. Graph based community detection is widely used for exploratory data analysis in many areas [43]. Representing the data as graph and performing the graph Laplacian enhances the clustering properties in the data. Such properties are generally not found in conventional clustering algorithms [44, 45]. For any type of data represented as a graph, community detection techniques can help in detecting the communities and their

hierarchical structures from within the data [46, 47]. These approaches have been used successfully for research problems in the fields of psychology, sociology, biology, statistics and computer science [46, 48, 49]. In this paper, we explore the use of community detection technique to mine structurally associated rules.

4. Network Communities based Hierarchical Lifelong Topic Modeling (NHLTM) Approach

In this section we present our proposed NHLTM approach. The lifelong learning aspect allows the model preserve rules of diverse nature over experience. The proposed community detection approach for mining rules facilitate in improving coherence of topics and arrange them into a hierarchical structure.

4.1. Architecture of NHLTM Approach

The Architecture that realizes the NHLTM approach is presented in Figure 1. A dataset is provided to this architecture as input. Hierarchically structured topics are produced from the given dataset as output. The rules mined from the datasets processed in the past contribute towards organizing words into topics and topics into a hierarchical structure. Our contribution in this research is to introduce a community detection based approach named NHLTM to mine rules with their inter-relations preserved. The architecture initiates the rule mining mechanism when new dataset is available as past data. The other modules i.e., rule representation and rule transfer are also slightly modified to support the efficient and effective utilization of the newly introduced community based rules. The knowledge base retains all the rules for long term future use while the maintenance module ensures the refinement of rules with new data.

4.2. Community Detection based Rule Mining

In order to perform community detection, the following four step process is generally used [45, 50]. First, represent the given data as a graph. Second, recursively split it into smaller and smaller communities until the stopping criteria is met. Generally, the communities at leaf nodes have strong correlation with exceptions of communities representing noise in the dataset. Third, the communities are evaluated to discard those that do not have enough compactness among nodes within the community. Fourth, only the communities

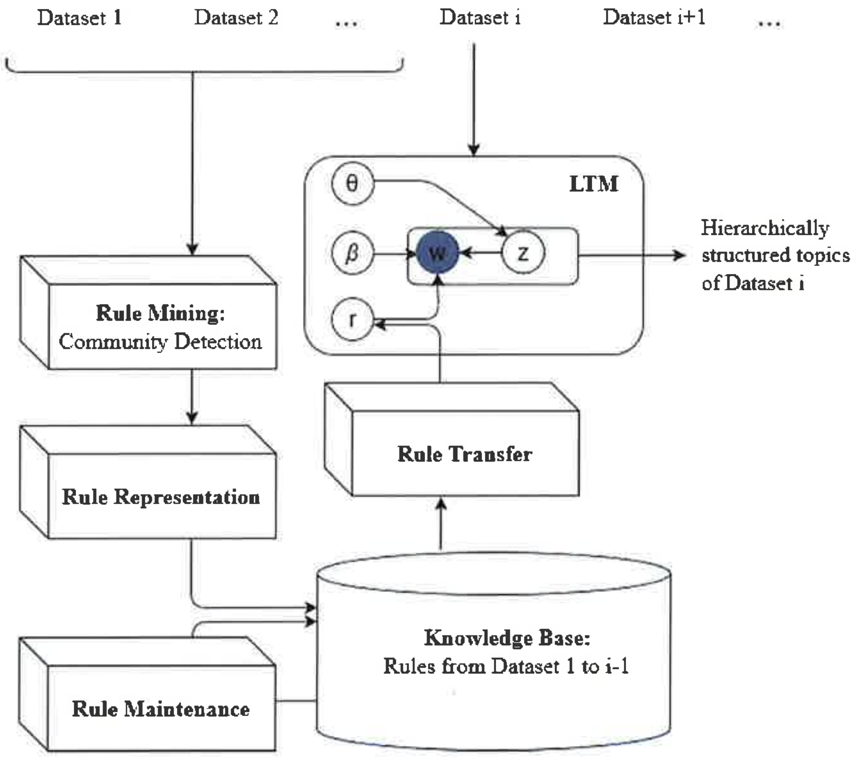

Figure 1: Architecture of NHLTM approach to produce hierarchically structured topics for Dataset i while utilizing the rules mined from the past i-1 Datasets

with acceptable correlation are retained. Our proposed NHLTM approach uses the aforementioned steps of community detection as the rule mining component. Subsequently, the rule representation component is also modified to represent the mined communities as rules. A better representation facilitate efficient and effective transfer of bias from rules into inference of LTM model.

Working of the proposed NHLTM approach is given in Algorithm 2. It takes the current Dataset i.e., $Dataseti$ as input while the past datasets $Datasets1toi-1$ are also available to it. A hierarchy of topics within $Dataseti$ is presented as output. Lines 1 - 3 present the working mechanism of the proposed NHLTM rule mining approach. Specifically, line 1 refers to the implementation of step 1 of community detection, i.e., to create a graph

Algorithm 1 Proposed NHLTM Approach

Input: Datasets $D = \{D^1, D^2, ..., D^i\}$

Output: Topic Hierarchy for D^i

1: Construct graph using Algorithm 2
2: Extract communities using Algorithm 3
3: Evaluate communities
4: Represent communities as rules
5: Sample relevant communities from i-1 tasks
6: LTM random initialization
7: **while** Words settle in their respective topics (Gibbs Sampling) **do**
8: Predict the most appropriate topic for the sampled word according to the current state
9: Update the word-topic and topic-document distributions accordingly
10: **end while**
11: Map rule associations to topics

from the available datasets. Line 2 is related to step 2 of community detection where we represents how the communities are identified and extracted. Line 3 represents step 3 of community detection that evaluates communities to retain only the communities with high intra-community associations. Line 4 relates to step 4 to represent communities as rules and retain them permanently. In line 5, rules that are relevant to *Dataseti* are considered. In line 6, the LTM model is randomly initialized. Lines 7 - 10 shows the mechanism of rule transfer into the LTM for the current dataset and relates to the final step of the approach. LTM uses Gibbs sampling as an inference technique to iteratively improve on the quality of topics. Further, the community detection process for rule mining is discussed in detail.

Step 1: Representing the data as graph

The first step to analyze this problem is to represent the available data as a graph. We construct Algorithm 2 for this purpose. It takes $i - 1$ datasets as input and generates a graph as output. The nodes represent words while the edges represent the co-occurrence of words within documents. The node for word w is represented as n_w while the edge between words w and w' is represented as $e_{w,w'}$, in lines 1 and 2. The strength of co-occurrence is represented with edge weight in line 3. In order to exploit all the available datasets, each dataset is consumed in creating the graph in lines 4 - 19. In lines 5 - 9, nodes are created for each unique word w in each dataset. In

This is too much detail. This alg is straight forward make this more concise

lines 6 - 8, a node n_w is checked if it already exists in the graph. For a node repeated across multiple datasets, continue to have a single node in the graph while its associations are accommodated from all the datasets. In lines 10 - 19, each document in each dataset is processed to add edges based on the co-occurrence of words within documents. Two words occurring in the same document i.e., $w \in d_j$ and $w' \in d_j$ is checked in lines 11 - 17. In line 11 we check if an edge $e_{w,w'}$ already exists between the words w and w'. In line 12, we increase its weight if it exists. If the edge does not exists, than in line 14, we create new edge with a weight being set to 1. Finally the graph is filtered by clipping weak branches in line 20 using equations,

$$weight(e_{w,w'}) = \begin{cases} \overline{weight}(e_{w,w'}) \times c_e & < \text{drop } e_{w,w'} \\ otherwise & retain \end{cases} \tag{3}$$

and

$$deg(n_w) = \begin{cases} \overline{deg}(n_w) \times c_n & < \text{drop } n_w \\ otherwise & retain \end{cases} \tag{4}$$

The threshold as the lower permissible limit to retain edges is set as $\overline{weight}(e_{w,w'}) \times c_e$, where $\overline{weight}(e_{w,w'})$ represents the average edge weight while c_e is the tolerance. Similarly, the threshold as the lower permissible limit to retain nodes is set as $\overline{deg}(n_w) \times c_n$, where $\overline{deg}(n_w)$ the average node degree while c_n represent tolerance. The advantage of dropping weak edges having low weights and weak nodes having low degrees is to reduce the computational cost. Since the graph is expected to grow in size, only reliable rules are extracted. The intuition for clipping weak branches is that they are not going to make strong rules due to their weak associations. They usually represent noise or rare vocabulary that is not frequently used by other users [45, 51]. Therefore, thresholds with typical values for c_e and c_n ranges between 0.2 and 0.33. These values are experimentally decided to drop out such nodes and edges [52].

Step 2: Identifying and Detecting word Communities

Communities can be extracted using different community detection algorithms [43, 44]. One way of extracting communities is to calculate Fiedler eigenvector. It represents the nodes with positive and negative values, indicating a possible split as the nodes of positive values may form one sub-community while the others may form another sub-community. It is obtained

Algorithm 2 Graph Construction

Input: Datasets of previously performed tasks $D = \{D^1, D^2, ..., D^{i-1}\}$

Output: Graph, g

```
n_w: Node of word w
e_{w,w'}: Edge between words w and w'
weight(e_{w,w'}): Weight of an edge signifies the co-occurrence association
    between words w and w'
for Dataset D^j ∈ D^{i-1} do
    for Word w in vocabulary of a Dataset D^j do
        if n_w ∉ g then
            g = g ∪ n_w
        end if
    end for
    for Document d_j in Dataset D^j do
        if w ∈ d_j and w' ∈ d_j and w ≠ w' then
            if e_{w,w'} exists then
                weight(e_{w,w'}) = weight(e_{w,w'}) + 1
            else
                weight(e_{w,w'}) = 1
            end if
        end if
    end for
end for
Trim the graph using Equations (3) and (4)
```

prose

by representing the graph g as a normalized Laplacian matrix and decomposing it into eigenvectors [53]. Graph cut weight (GCW) evaluates if it is feasible to proceed with splitting the graph into communities and sub-communities recursively. The evaluation is based on the cost associated as inter-community associations. Repeating the process i.e., finding possible splits through Fiedler vector and evaluating them through GCW iteratively cut communities in two halves. A stopping condition is met when all the splits are too expensive to proceed with.

The community extraction mechanism is demonstrated in Algorithm 3. It takes a graph as input and produce hierarchical communities as output. Lines 1 - 13 define a procedure named *split_communities* that is used to split a community into two. It takes a community as argument with the graph

prose

Algorithm 3 Extracting hierarchical communities from graph

Input: Graph g
Output: Hierarchical Communities

1: **procedure** *split_communities*(comm = g)
2: Calculate Normalized Laplacian for comm using Eq. (5) and (6)
3: Calculate Fiedler vector for *comm*
4: Find two communities $comm_a$ and $comm_b$ as cut on *comm*
5: **if** GraphCutWeight($comm_a$, $comm_b$) $\leq c_{GCW}$ **then**
6: Split *comm* into $comm_a$ and $comm_b$
7: Preserve the associations between $comm_a$ and $comm_b$
8: *split_communities*($comm_a$)
9: *split_communities*($comm_b$)
10: **else**
11: stop
12: **end if**
13: **end procedure**

being considered as default community in first call. In order to process a community for possible separation into two smaller communities, we consider a normalized Laplacian in line 2 which is computed as,

not needed well known.

$$\mathcal{L}(w, w') = \begin{cases} 1 & if w = w' \wedge deg(w) = deg(w') \neq 0 \\ -\frac{1}{\sqrt{deg(w) \times deg(w')}} & if e_{w,w'} \in E \\ 0 & otherwise \end{cases} \quad (5)$$

that gives matrix representation to a community for studying its many useful properties. Sparsest cut is one such property that suggests the separation of vertices into two smaller communities with minimum edges across the cut. The Laplacian can also be represented in the form of a matrix as,

not needed.

$$L^{sym} = D^{-1/2} L D^{1/2} = I - D^{-1/2} A D^{-1/2} \quad (6)$$

Where I is an identity matrix, A is an adjacency matrix and D is a diagonal matrix. The eigenvalue decomposition of the normalized Laplacian gives eigenvectors where the eigenvector corresponding to the second smallest eigenvalue is called Fiedler vector, in line 3. It holds positive values for some

nodes while negative for others. The two types of nodes indicate the two possible communities to be formed as $comm_a$ and $comm_b$, in line 4. Lines 5 - 10 verifies if the cost of splitting $comm$ into $comm_a$ and $comm_b$ is within the threshold c_{GCW}. Graph cut weight (GCW) for $comm_a$ and $comm_b$ can be calculated as,

$$GCW(comm_a, comm_b) = \frac{\sum_{w \in comm_a} (\sum_{w' \in comm_b} weight(e_{w,w'}))}{\sum_{w} (\sum_{w'} weight(e_{w,w'}))}. \tag{7}$$

The sign of the Fiedler eigenvector can be used to cut the graph into two communities as two halves of vertices having positive and negative values respectively. The process stops when the suggested cut is expensive that is having many edges across it, in line 11. In line 6, $comm_a$ and $comm_b$ are transformed into individual communities while their inter-community associations are preserved in line 7. It has been previously ignored by the existing approaches. Lines 8 and 9 recursively call the *split_communities* procedure to repeat the process for the newly formed communities.

Step 3: Evaluating Compactness of Communities

The communities extracted in step 2 are evaluated for their quality in terms of associations among the nodes of the community, using Algorithm 4. For each community $comm_i \in comm$ is iterated in lines 1 - 7 and their $WGCI$ is calculated as,

$$WGCI(comm_i) = \sum_{e_{w,w'}} \frac{weight(e_{w,w'})}{\sqrt{deg(w) \times deg(w')}}. \tag{8}$$

It measures the intra-community associations in relation to the inter-community relations. Nodes with higher intra-community associations have higher $WGCI$ score as the intra-community edge weights increases proportional to the degree of nodes. In contrast, the nodes with high inter-community associations have low Weighted graph connectivity index $WGCI$ as the intra-community edges will have low weights and their respective nodes have high degrees. Von Neumann entropy is also used for evaluating communities as,

Algorithm 4 Evaluating and filtering communities with weak intra-community associations

Input: All communities $comm$

Output: Selected communities $comm_{selected} \subset comm$

1: **for** each $comm_i$ **do**
2:     **if** (WGCI($comm_i$) $> c_{WGCI}$) or (entropy($comm_i$) $> c_{entropy}$) **then**
3:         $comm_{selected} = comm_{selected} \cup comm_i$
4:     **else**
5:         remove $comm_i$
6:     **end if**
7: **end for**

$$Entropy(S) = -\sum_{n=1}^{|N|} \frac{\hat{\lambda}_n}{2} ln \frac{\hat{\lambda}_n}{2} \tag{9}$$

where N is the total number of nodes in the community and λ_n represents their eigenvalues of the normalized adjacency matrix corresponding to the second lowest value. Two thresholds c_{WGCI} and $c_{entropy}$ are defined to select communities that have considerable associations among its nodes. The $WGCI$ and entropy of each community are compared to these thresholds in lines 2 - 6. The communities that satisfy these thresholds are preserved as $comm_{selected}$, while others are discarded in line 5.

The threshold values are experimentally chosen to keep only those communities as rules that have higher intra-community associations [54, 55]. Such rules have higher impact in grouping the words of a rule together within a topic. The communities with lower intra-community associations may also be retained but they have lower impact and therefore, cannot effectively influence the inference technique to help in grouping its words together as a topic. Therefore, in order to reduce the computational cost, such communities are ignored and are not retained as rules.

Step 4: Representing Communities as Rules

The communities selected are abstracted and represented as rules. The objective is to facilitate efficient storage, retrieval and transfer of rules into a task [56]. The words of the community are retained as the rule words which also serve as the context of the rule. The structural information of a

community is retained in the rule by preserving the order of splits that lead to the generation of each community. The impact of a rule is calculated using Lins Approximation which is the quadratic approximation of Von Neumann entropy [44] as,

$$LinsApproximation = \frac{|N|}{4} - \sum_{e_{w,w'}} \frac{1}{4deg(w) \times deg(w')}. \tag{10}$$

The weights of all edges in the community are added up and normalized by dividing with the square root of the product of degree of two nodes $deg(w)$ and $deg(w')$ that are sharing the edge. N represents the total number of nodes in the community. It is aggregated across all edges of the community to give a measure of entropy for the community evaluated.

Final Step: Transferring the impact of rules into gibbs sampling

This component deals with transferring the impact of relevant rules into the gibbs sampling inference of topic distribution for the current task. The default gibbs sampling inference technique continuously update the document-topic and topic-word distributions. It starts from a random state and converges on the suitable distribution in iterations [16]. These distributions in any iteration refer to the state of the model, where new state is determined based on the previous state following markov chain. It can be represented as,

$$P(z_i = t|z^{-i}, w, d, \alpha, \beta) \propto \theta_{d,t} \times \phi_{t,w} \tag{11}$$

where a sampled word w from document d has its probability updated for topic t as $\theta_{d,t}$ times $\phi_{t,w}$. While, $\theta_{d,t}$ and $\phi_{t,w}$ are represented as,

$$\theta_{d,t} = \frac{\eta_{d,t}^{-i} + \alpha}{\sum_{k=1}^{T}(\eta_{d,k}^{-i} + \alpha)} \tag{12}$$

and

$$\phi_{t,w} = \frac{\eta_{t,w}^{-i} + \beta}{\sum_{u=1}^{V}(\eta_{t,u}^{-i} + \beta)} \tag{13}$$

respectively. In the Eq.(12, the document-topic distribution i.e., $\theta_{d,t}$ is updated using $\eta_{d,t}^{-i}$ as the probability of topic t in document d based on the

current state of the model. It is calculated excluding the sampled occurrence of the word, indicated by $-i$. It uses α as the smoothing factor. The denominator normalizes the value using the sum of probabilities of all the other topics from $k = 1, ..., T$ for the same document d. Similarly in Eq.(13, the topic-word distribution i.e., ϕ is updated using $\eta_{t,w}^{-i}$ as the probability of word w in topic t based on the current state of the model. It is again calculated excluding the sampled occurrence of the word w, indicated by $-i$. It uses β as the smoothing factor. The denominator normalizes the value using the sum of probabilities of all the words from $u = 1, ..., V$ for the same topic t.

In order to select relevant rules for a task, the overlap of a rule is measured with the vocabulary of the task and are compared against threshold. It is presented using equation from [11] as,

$$\frac{\#(V \cap r)}{\#r} > \xi_{overlap} \tag{14}$$

where $\xi_{overlap}$ is the threshold for overlap between rule words and the vocabulary of the current task. It helps to resolve the context and address the word sense disambiguation for a rule.

The bias from the rules effects the topic-word distribution i.e., $\phi_{t,w}$ only. When the probability of a word w is increased for a topic t and w is part of a rule $w \in r$, then the other words of the rule $w' \in r$ also have their probabilities increased for the same topic t, using equation,

$$\phi_{t,w',r} = \frac{\nu_{r_{w,w' \in r}} \times \eta_{t,w'}^{-i} + \beta}{\sum_{u=1}^{V} (\nu_{r_{w,w' \in r}} \times \eta_{t,u=w'}^{-i} + \beta)} \tag{15}$$

where the probabilities of all other words $w' \in r$ are updated for topic t by the ν_r factor of the rule when $w \in r$ has its probability updated for t. However, in case $w \notin r$, the default Eq. (13) will be used to update $\phi_{t,w}$.

5. Experimental Results and Discussion

In this section, we present detailed experimental results of the proposed NHLTM model for extracting topic hierarchies for a sequence of tasks. The standard Chen2014 dataset is considered for this purpose. This dataset is specifically used for lifelong topic modeling in literature. It has a collection of 100 datasets where 50 are about electronic products while the other half is about non-electronic products. Each dataset has more than 5000 commercial

Only one dataset?!

Table 4: Complex Network Analysis, for N tasks

Property	Before filtering Value	After filtering Value
Total Vertices:	5,574	1,228
Total Edges	6,55,261	3,46,687
Total Edge Weight:	21,90,564	16,81,148

product reviews as documents, collected from Amazon.com. Therefore, it fits more into the context of mining rules continuously as an automatic process. Lifelong topic modeling cannot be applied to a single dataset as it requires multiple tasks to improve with experience.

For the sake of experiment, we used the 50 electronic product datasets. Only 10% of the past data is considered for mining rules to reduce computational cost. Table 4 shows the details of the graph having a total of 5,574 nodes i.e, unique words. Please note that the dataset is preprocessed to retain only those parts-of-speech that may represent a topic i.e., verb, adverb, adjective and noun. The nodes or vertices are interlinked with each other sharing a total of 6,55,261 unique edges. On average there are 117.55 unique edges per node. The average weight of an edge is 3.34. The last value in Table 4 is the total edge weight as the measure of sum of all edge weights and is 21,90,564. It means that there are many edges representing weak associations between the words. Therefore aggressive filtering may be needed for filtering out such nodes/edges.

Filtering out edges with smaller weights and nodes with fewer edges, the graph is refined for efficient analysis. The filtering mechanism has dropped the total nodes of the network to 1,228 while the total edges are reduced to 3,46,687 which improves the per node edges on average to 282.318. Similarly the total co-occurrences among all nodes is 1,681,148 which has the average edge weight of a node improved to 4.859. Thus we have a smaller but more densely connected network after the filtering phase.

In the second step, the graph is processed to identify and extract possible communities. There are a total 148 communities extracted that are interrelated to each other through structural associations. Structures are preserved through iteratively splitting the communities into sub-communities. The communities that are separated in later iterations are considered closer as compared to the communities that are separated in the former iterations. The structural position of each community in the graph of all previous datasets

Table 5: Selected communities learnt from 50 tasks

Comm Sr.No.	Structural Info. (Hexadecimal Codes)	Top Words
1	9	Adaptor, cam, filter, stand, indicator
2	C	Exception, weird, superior, break
3	56	Field, section, flat
4	8A	Storage, airport, convenient
5	21E	Environment, rest,world, answer
6	23D	Folder, label, credit
7	439	Profile, background, scene, rock
8	43B	Tray, blueray, manual, gripe
9	10A9	Alarm, longer, beep
10	2146	Police, ticket, state, ka
11	2151	Dead, transmitter, awful
12	8528	Hard, top, lap, scroll, pad
13	10A63	Outstanding, playback, release, model
14	10A70	Gamer, thrive bigger, hub
15	10B3E	Toshiba, lifetime, replacement, warranty
16	21091	Foot, pace, distance, run
17	79A0D	Heavy, metal, cover, bottom
18	10C0C3	Traffic,highway,city, road
19	214C93	Bluetooth, reception, works, feedback
20	214C90	Bright, display, control

is derived through subsequent splits and are presented as hexadecimal codes in Table 5. The communities with smaller difference of hexadecimal codes indicate a nearer common parent and those a closer relationship and vice versa. Figure 2 shows the first five iterations of recursively splitting communities into sub-communities. The communities with grey background will continue with the process. The others with white background have reached their stopping criteria of either too few nodes or too expensive cut to continue with.

In the third step, the communities extracted are evaluated for their quality. Communities with weakly associated nodes cannot effectively communicate their impact and are therefore filtered out. Only 84 communities were left behind after the *WGCI* and entropy based filtering. The threshold values for *WGCI* and entropy were set to 1 and 0.2, respectively. These values are experimentally evaluated for the given dataset. Dropping the thresholds add to computational cost without showing reasonable improvement in results. Table 5 shows the structural information and the top few words

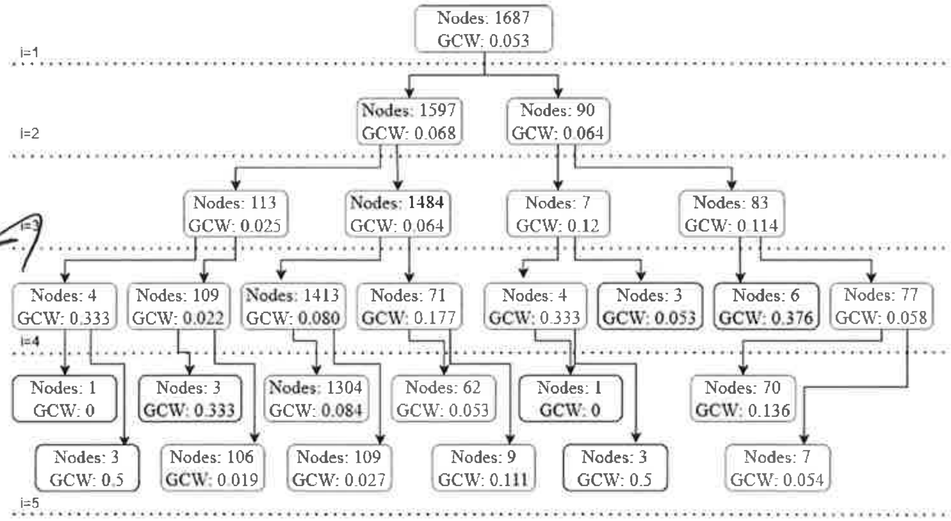

Figure 2: Hierarchical breakdown of graph into communities till first 5 levels, nodes per community and their respective Graph cut weight.

for 20 sample rules. Table 6 shows other properties i.e., size, entropy, Lins approximation, $WGCI$ and average degree node across community for the corresponding rules in Table 5.

In the fourth step, the Lins Approximation is used as the impact of a rule. It refers to the bias with which the inference technique is manipulated in favor of the rule by using generalized polya urn model. It can be observed that most of the rules have their sizes ranging from 4 to 7 nodes as the natural arrangement of words in the given data. It may vary for other datasets and therefore, the learning mechanism is kept flexible to adapt to it.

The quality of the topics extracted is evaluated using topic coherence. It is the dominant topic evaluation technique that has the highest relevance with human judgment. A higher value indicates better correlation among the words of a topic. The learning curve of lifelong topic modeling is evaluated based on improvement in the quality of topics as topic coherence. The topic coherence of NHLTM approach is compared with state-of-the art lifelong topic models including LTM, AMC-M, AMC and OAMC. Top 30 words in a topic are used to calculate the topic coherence of a topic as in literature. The proposed approach gives better topic coherence in comparison to the existing approach which suggests the usefulness of the rules mined through community detection. The results are also depicted in Figure 3.

Table 6: Properties of the communities shown in Table 5

Comm Sr.No.	Size	Entropy	Lins Approx.	$WGCI$	Avg. Node Degree
1	7	2.787	1.8	7.073	56.893
2	13	3.816	3.249	0.236	12.153
3	5	1.108	1.249	0.020	2
4	5	1.130	1.249	0.040	4.6
5	6	1.440	1.499	0.040	3.83
6	5	1.055	1.249	0.030	2.4
7	8	2.087	1.999	0.061	4.125
8	5	1.085	1.249	0.054	6.6
9	6	1.425	1.499	0.86	7.83
10	5	1.135	1.249	0.336	19.2
11	6	1.449	1.499	0.036	3.33
12	28	1.745	1.749	0.0833	6.85
13	6	1.488	1.499	0.0923	10.83
14	5	1.109	1.249	0.0414	3.8
15	5	1.071	1.249	0.3227	48.6
16	7	1.698	1.749	1.538	103.285
17	5	1.129	1.249	0.348	52.8
18	7	1.817	1.749	0.829	52.85
19	5	1.141	1.249	0.062	6
20	4	1.746	1.448	0.085	75.4

A hierarchy of topics is extracted for each dataset that shows the concepts discussed within the dataset at different granularity levels. Table 7 provides a comparison of topic hierarchies generated by the proposed NHLTM approach and HLDA approach for the Alarm clock dataset. The hierarchies are compared in terms of their topic coherence at different levels in the hierarchy as the number of topics increases. The empirical log-likelihood indicates generality of the models as its ability to estimate the unseen data. Topic coherence of the proposed NHLTM approach increases from -1024 for level one to -804 for the topics of level six. The topic coherence of HLDA increases from -1143 for level one to -807 for the topics of the sixth level. The topics at higher levels represent generic concepts and therefore, attain lower topic coherence. While the lower levels have specific concepts with compact representation. The given results indicate compactness of topics produced by NHLTM at all levels of the hierarchy. The value of empirical log-likelihood should preferably increase or stabilize as the model makes better prediction of the unseen data. The empirical log-likelihood values produced by NHLTM

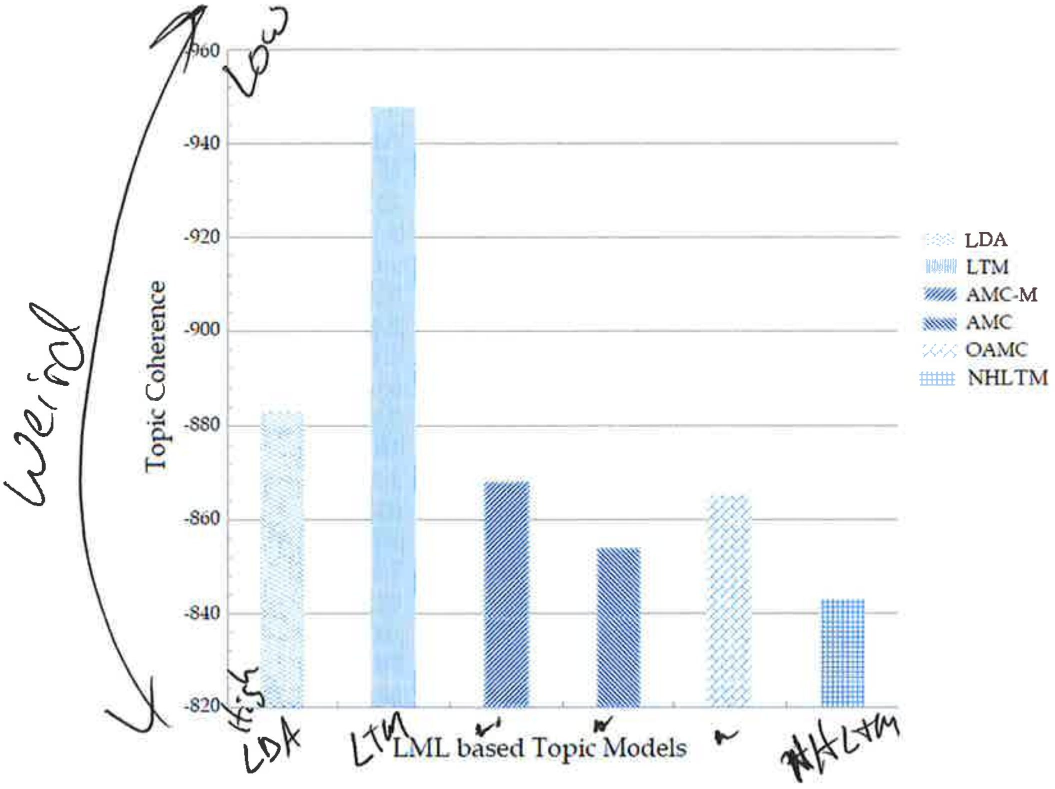

Figure 3: Comparison of topic coherence of lifelong topic models for 50 datasets

model are maintained between -115 and -113, whereas that of HLDA is between -1[illegible]4 and -120. It is also depicted in the Figure 4.

The topics of the *Alarm Clock* dataset are more scattered having more branches at the lower level in Figure 5. A possible reason is that the users discussing topics of *Alarm Clock* referred to the same topics using a variety of expressions. Discussing more topics within a document leads a closer relation among those topics. The topics *Noise wake* and *Design flaw* have very weak association with all the other topics and are therefore, directly linked to the root at the highest level. The root represents the domain of the dataset, which is *Alarm Clock* in this case. The topics *Small bedside* and *Easy cheap* are closely related. It broadly refers to the comfort level that the *Alarm Clock* is cheap, its easy available, its small in size and can be easily placed on the bedside. The other topics that are closely related,

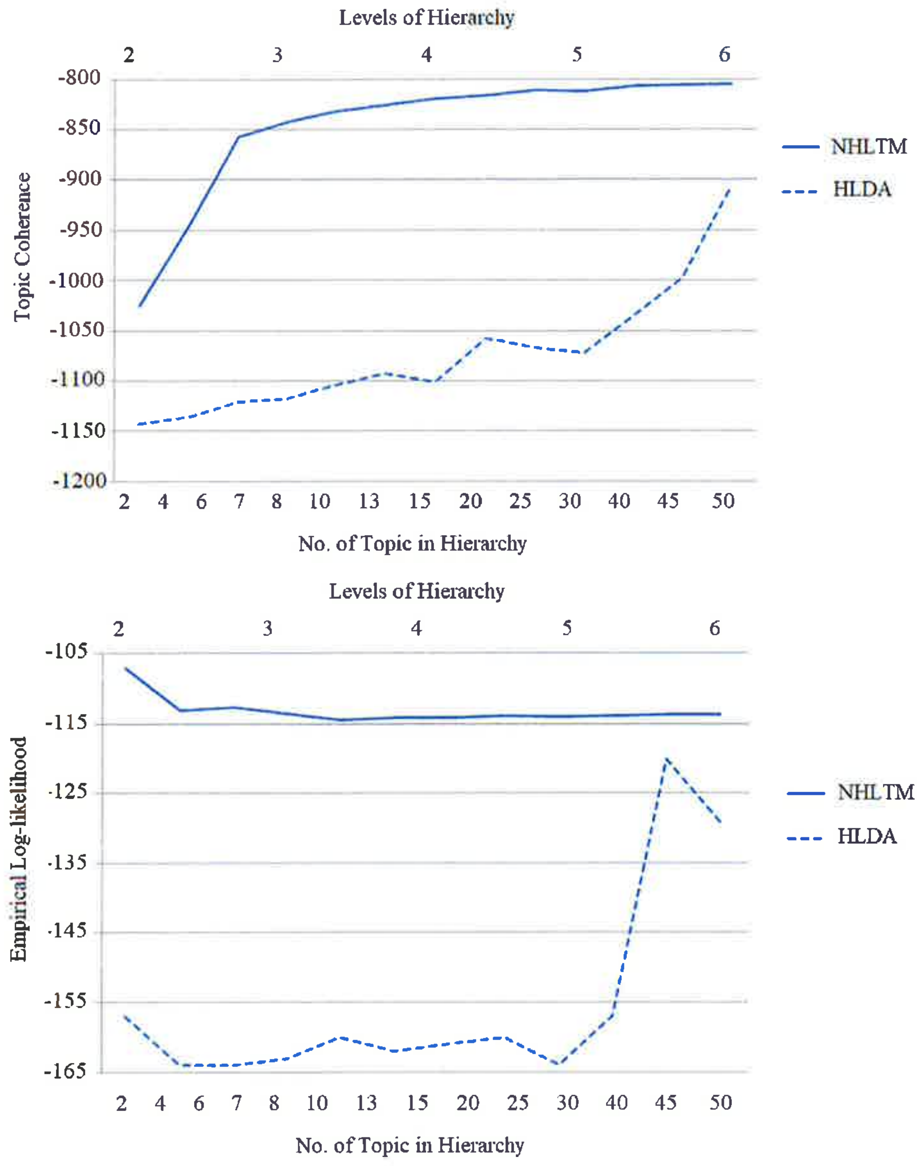

Figure 4: Comparison of NHLTM and HLDA with increasing number of topics and levels of hierarchy for the Alarm clock dataset in large-scale data

Table 7: Comparison of topic hierarchies produced by NHLTM and HLDA using topic coherence and empirical log-likelihood for increasing topics at different levels of hierarchy

Topics	Level	NHLTM TC	HLDA TC	NHLTM ELL	HLDA ELL
2	1	-944	-1136	-106	-129
4	2	-856	-1121	-113	-120
6	3	-842	-1118	-112	-157
7	4	-832	-1104	-113	-164
8	5	-825	-1093	-114	-160
10	6	-819	-1101	-114	-161
13	7	-816	-1058	-114	-162
15	8	-810	-1067	-113	-160
20	9	-812	-1073	-113	-163
30	12	-806	-1036	-113	-164
40	14	-805	-998	-113	-160
50	15	-804	-954	-113	-157

represent the operational topics as *Light feature, Simple stuff* and *Digital number*. The topics *Radio instructions* and *Display control* represent the soft features or controls of the product. These topics are linked to the previous group of topics as operations and controls are somewhat closely related. The topics *Battery star* and *Plastic piece* doesn't have a very clear sense but loosely represent the exterior of the product. The next few topics also have weaker association. The non-electronic products like *Cars, Boats* and *Food* would have more diverse vocabulary as compared to electronic products and therefore, their topic hierarchies could be more easily comprehendible.

6. Conclusion

Lifelong machine learning enables topic models to automatically extract rules and utilize them to improve the quality of topics for an unknown dataset. Presenting the topics as a multiple level hierarchy allows the users to focus on the specialized topics for deep analysis without losing their relevance to few generic topics in a structure. It facilitates to move up and down the hierarchy depending upon the complexity or details that can be handled. The existing lifelong topic modeling approaches group words into topics but do not retain structural information for extracting topic hierarchies. The proposed community detection based rule mining approach and the NHLTM architecture that supports it, helps to mine structurally associated rules that

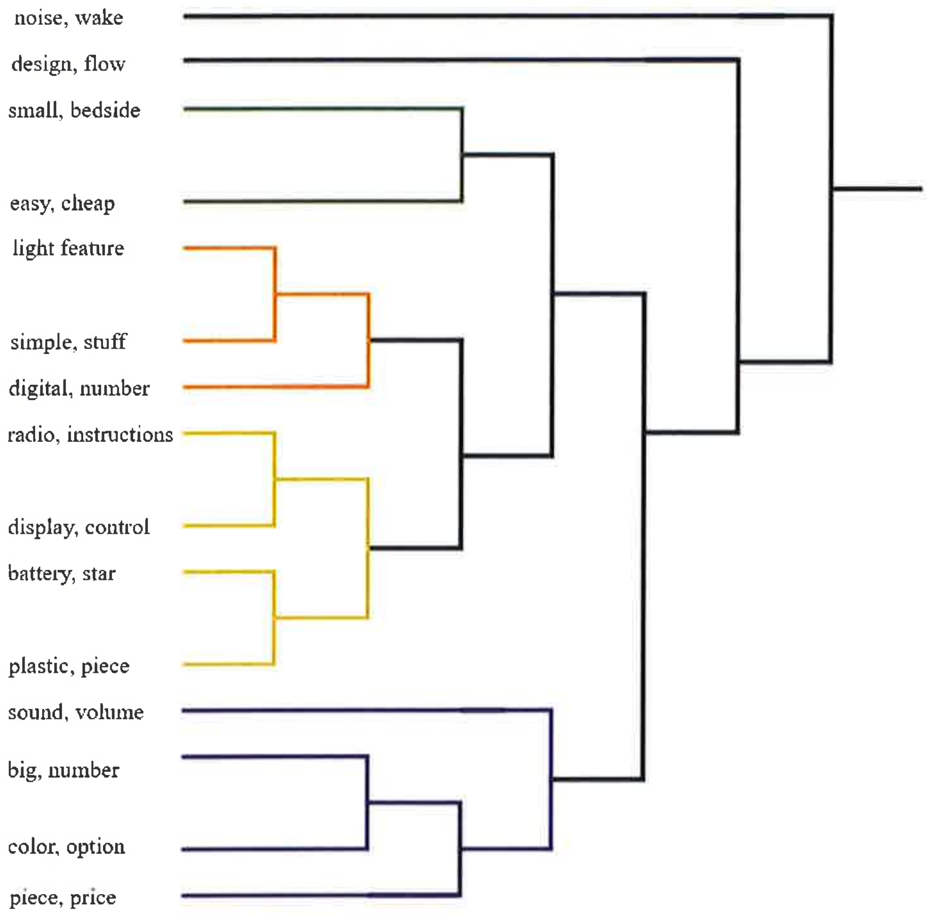

Figure 5: Topic Hierarchy of Alarm Clock Dataset

make them available for use in lifelong topic modeling. The intra-rule associations among words help in improving the quality of topics while the inter-rule word associations help in generating the hierarchical structure of topics. Experimental results suggest that the proposed approach improves topic coherence from -.836 for generic topics to .804 for specific topics which is better by 12 and 40 points comparing to AMC and baseline-LDA, respectively.

7. Acknowledgements

This work is partially supported by faculty research support fund of National University of Computer and Emerging Sciences (NUCES), Pakistan.

References

[1] T. Wang, Y. Cai, H.-f. Leung, R. Y. Lau, Q. Li, and H. Min, "Product aspect extraction supervised with online domain knowledge," *Knowledge-Based Systems*, vol. 71, pp. 86–100, 2014.

[2] X. Zheng, Z. Lin, X. Wang, K.-J. Lin, and M. Song, "Incorporating appraisal expression patterns into topic modeling for aspect and sentiment word identification," *Knowledge-Based Systems*, vol. 61, pp. 29–47, 2014.

[3] P. Ruvolo and E. Eaton, "Ella: An efficient lifelong learning algorithm.," *Proceedings of the 30th International Conference on Machine Learning (ICML-13)*, vol. 28, pp. 507–515, 2013.

[4] S. Wang, M. Zhou, S. Mazumder, B. Liu, and Y. Chang, "Disentangling aspect and opinion words in target-based sentiment analysis using lifelong learning," *arXiv preprint arXiv:1802.05818*, 2018.

[5] X. Zhang and R. He, "Topic extraction of events on social media using reinforced knowledge," in *International Conference on Knowledge Science, Engineering and Management*, pp. 465–476, Springer, 2018.

[6] D. L. Silver, Q. Yang, and L. Li, "Lifelong machine learning systems: Beyond learning algorithms.," in *Association for the Advancement of Artificial Intelligence AAAI Spring Symposium: Lifelong Machine Learning*, pp. 49–55, Citeseer, 2013.

[7] A. Mukherjee and B. Liu, "Aspect extraction through semi-supervised modeling," in *Proceedings of the 50th Annual Meeting of the Association for Computational Linguistics: Long Papers-Volume 1*, pp. 339–348, Association for Computational Linguistics, 2012.

[8] M. T. Khan, N. Azam, S. Khalid, and J. Yao, "A three-way approach for learning rules in automatic knowledge-based topic models," *International Journal of Approximate Reasoning*, vol. 82, pp. 210–226, 2017.

[9] X.-L. Mao, Z.-Y. Ming, T.-S. Chua, S. Li, H. Yan, and X. Li, "Sshlda: a semi-supervised hierarchical topic model," in *Proceedings of the 2012 Joint Conference on Empirical Methods in Natural Language Processing and Computational Natural Language Learning*, pp. 800–809, Association for Computational Linguistics, 2012.

[10] D. Andrzejewski, X. Zhu, and M. Craven, "Incorporating domain knowledge into topic modeling via dirichlet forest priors," in *Proceedings of the 26th Annual International Conference on Machine Learning*, pp. 25–32, ACM, 2009.

[11] M. T. Khan, M. Durrani, S. Khalid, and F. Aziz, "Online knowledge-based model for big data topic extraction," *Computational intelligence and neuroscience*, vol. 2016, 2016.

[12] G. Bouma, "Normalized (pointwise) mutual information in collocation extraction," *Proceedings of German Society of Computational Linguistics & Language Technology (GSCL)*, pp. 31–40, 2009.

[13] D. M. Blei, T. L. Griffiths, and M. I. Jordan, "The nested chinese restaurant process and bayesian nonparametric inference of topic hierarchies," *Journal of the ACM (JACM)*, vol. 57, no. 2, p. 7, 2010.

[14] E. Kamar, A. Kapoor, E. Horvitz, and W. Redmond, "Lifelong learning for acquiring the wisdom of the crowd.," in *International Joint Confernece on Artificial Intelligence (IJCAI)*, vol. 13, pp. 2313–2320, Citeseer, 2013.

[15] Z. B. Chen, "chen2014 dataset," https://www.cs.uic.edu/ zchen/downloads/KDD2014-Chen-Dataset.zip, 2014. Accessed: 2018-01-08.

[16] D. M. Blei, A. Y. Ng, and M. I. Jordan, "Latent dirichlet allocation," *Journal of Machine Learning Research*, vol. 3, pp. 993–1022, 2003.

[17] Q. Mei, X. Ling, M. Wondra, H. Su, and C. Zhai, "Topic sentiment mixture: modeling facets and opinions in weblogs," in *Proceedings of the 16th international conference on World Wide Web*, pp. 171–180, ACM, 2007.

[18] J. Huang, M. Peng, and H. Wang, "Topic detection from large scale of microblog stream with high utility pattern clustering," in *Proceedings of*

the 8th Workshop on Ph. D. Workshop in Information and Knowledge Management, pp. 3–10, ACM, 2015.

[19] C. Lin, Y. He, R. Everson, and S. Rüger, "Weakly supervised joint sentiment-topic detection from text," *IEEE Transactions on Knowledge and Data Engineering*, vol. 24, no. 6, pp. 1134–1145, 2012.

[20] M. T. Khan and S. Khalid, "Sentiment analysis for health care," *International Journal of Privacy and Health Information Management (IJPHIM)*, vol. 3, no. 2, pp. 78–91, 2015.

[21] S. Branavan, H. Chen, J. Eisenstein, and R. Barzilay, "Learning document-level semantic properties from free-text annotations," *Journal of Artificial Intelligence Research*, vol. 34, pp. 569–603, 2009.

[22] F. Li, M. Huang, and X. Zhu, "Sentiment analysis with global topics and local dependency.," in *Association for the Advancement of Artificial Intelligence (AAAI)*, vol. 10, pp. 1371–1376, 2010.

[23] D. Ramage, D. Hall, R. Nallapati, and C. D. Manning, "Labeled lda: A supervised topic model for credit attribution in multi-labeled corpora," in *Proceedings of the Empirical Methods in Natural Language Processing, Volume 1*, pp. 248–256, 2009.

[24] J. D. Mcauliffe and D. M. Blei, "Supervised topic models," in *Advances in neural information processing systems*, pp. 121–128, 2008.

[25] J.-H. Kang, J. Ma, and Y. Liu, "Transfer topic modeling with ease and scalability.," in *The 12th Society for Industrial and Applied Mathematics SIAM International Conference on Data Mining*, pp. 564–575, SIAM, 2012.

[26] G.-R. Xue, W. Dai, Q. Yang, and Y. Yu, "Topic-bridged plsa for cross-domain text classification," in *Proceedings of the 31st annual international ACM Special Interest Group on Information Retrieval SIGIR conference on Research and development in information retrieval*, pp. 627–634, ACM, 2008.

[27] D. Andrzejewski, X. Zhu, M. Craven, and B. Recht, "A framework for incorporating general domain knowledge into latent dirichlet allocation

using first-order logic," in *IJCAI Proceedings-International Joint Conference on Artificial Intelligence*, vol. 22, p. 1171, 2011.

[28] Z. Chen, A. Mukherjee, B. Liu, M. Hsu, M. Castellanos, and R. Ghosh, "Discovering coherent topics using general knowledge," in *Proceedings of the 22nd ACM international conference on Information & Knowledge Management*, pp. 209–218, ACM, 2013.

[29] Z. Chen and B. Liu, "Topic modeling using topics from many domains, lifelong learning and big data," in *Proceedings of the 31st International Conference on Machine Learning (ICML-14)*, pp. 703–711, 2014.

[30] Y. W. Teh, M. I. Jordan, M. J. Beal, and D. M. Blei, "Hierarchical dirichlet processes," *Journal of the american statistical association*, 2012.

[31] Z. Chen and B. Liu, "Mining topics in documents: standing on the shoulders of big data," in *Proceedings of the 20th ACM SIGKDD international conference on Knowledge discovery and data mining*, pp. 1116–1125, ACM, 2014.

[32] R. Raina, A. Battle, H. Lee, B. Packer, and A. Y. Ng, "Self-taught learning: transfer learning from unlabeled data," in *Proceedings of the 24th international conference on Machine learning*, pp. 759–766, ACM, 2007.

[33] Z. Chen, A. Mukherjee, and B. Liu, "Aspect extraction with automated prior knowledge learning," in *52nd Annual Meeting of the Association for Computational Linguistics*, pp. 347–358, 2014.

[34] E. Eaton and P. L. Ruvolo, "Ella: An efficient lifelong learning algorithm," in *Proceedings of the 30th international conference on machine learning (ICML-13)*, pp. 507–515, 2013.

[35] Z. Chen and B. Liu, "Lifelong machine learning," *Synthesis Lectures on Artificial Intelligence and Machine Learning*, vol. 10, no. 3, pp. 1–145, 2016.

[36] K. W. Church and P. Hanks, "Word association norms, mutual information, and lexicography," *Computational linguistics*, vol. 16, no. 1, pp. 22–29, 1990.

[37] D. Andrzejewski and X. Zhu, "Latent dirichlet allocation with topic-in-set knowledge," in *Proceedings of the North American Chapter of the Association for Computational Linguistics: Human Language Technologies NAACL: HLT 2009 Workshop on Semi-Supervised Learning for Natural Language Processing*, pp. 43–48, Association for Computational Linguistics, 2009.

[38] S. Kim, J. Zhang, Z. Chen, A. H. Oh, and S. Liu, "A hierarchical aspect-sentiment model for online reviews.," in *Association for the Advancement of Artificial Intelligence (AAAI)*, 2013.

[39] D. Griffiths and M. Tenenbaum, "Hierarchical topic models and the nested chinese restaurant process," *Advances in neural information processing systems*, vol. 16, p. 17, 2004.

[40] M. T. Khan, S. Yar, S. Khalid, and F. Aziz, "Evolving long-term dependency rules in lifelong learning models," in *Knowledge Engineering and Applications (ICKEA), IEEE International Conference on*, pp. 93–97, IEEE, 2016.

[41] M. Xu, R. Yang, S. Harenberg, and N. F. Samatova, "A lifelong learning topic model structured using latent embeddings," in *Semantic Computing (ICSC), 2017 IEEE 11th International Conference on Semantic Computing*, pp. 260–261, IEEE, 2017.

[42] S. Wang, Z. Chen, and B. Liu, "Mining aspect-specific opinion using a holistic lifelong topic model," in *Proceedings of the 25th International Conference on World Wide Web*, pp. 167–176, International World Wide Web Conferences Steering Committee, 2016.

[43] U. Von Luxburg, "A tutorial on spectral clustering," *Statistics and computing*, vol. 17, no. 4, pp. 395–416, 2007.

[44] L. Han, F. Escolano, E. R. Hancock, and R. C. Wilson, "Graph characterizations from von neumann entropy," *Pattern Recognition Letters*, vol. 33, no. 15, pp. 1958–1967, 2012.

[45] N. Du, B. Wang, and B. Wu, "Community detection in complex networks," *Journal of Computer Science and Technology*, vol. 23, no. 4, pp. 672–683, 2008.

[46] F. Escolano, E. R. Hancock, and M. A. Lozano, "Heat diffusion: Thermodynamic depth complexity of networks," *Physical Review E*, vol. 85, no. 3, p. 036206, 2012.

[47] K. Benzi, B. Ricaud, and P. Vandergheynst, "Principal patterns on graphs: Discovering coherent structures in datasets," *IEEE Transactions on Signal and Information Processing over Networks*, vol. 2, no. 2, pp. 160–173, 2016.

[48] S. Peng, A. Yang, L. Cao, S. Yu, and D. Xie, "Social influence modeling using information theory in mobile social networks," *Information Sciences*, vol. 379, pp. 146–159, 2017.

[49] D. Simmie, N. Thapen, and C. Hankin, "Defender: Detecting and forecasting epidemics using novel data-analytics for enhanced response," *arXiv preprint arXiv:1504.04357*, 2015.

[50] A. JS and M. Kaimal, "Community detection in complex networks," pp. 1–13, 2014.

[51] L. Danon, A. Diaz-Guilera, J. Duch, and A. Arenas, "Comparing community structure identification," *Journal of Statistical Mechanics: Theory and Experiment*, vol. 2005, no. 09, p. P09008, 2005.

[52] F. Aziz, E. R. Hancock, and R. C. Wilson, "Graph entropy from closed walk and cycle functionals," in *Joint IAPR International Workshops on Statistical Techniques in Pattern Recognition (SPR) and Structural and Syntactic Pattern Recognition (SSPR)*, pp. 174–184, Springer, 2016.

[53] B. Li, Y.-R. Li, and X.-L. Zhang, "A survey on laplacian eigenmaps based manifold learning methods," *Neurocomputing*, 2018.

[54] L. Massoulié, "Community detection thresholds and the weak ramanujan property," in *Proceedings of the forty-sixth annual ACM symposium on Theory of computing*, pp. 694–703, ACM, 2014.

[55] J. Banks, C. Moore, J. Neeman, and P. Netrapalli, "Information-theoretic thresholds for community detection in sparse networks," in *Conference on Learning Theory*, pp. 383–416, 2016.

[56] L. Sang, M. Xu, S. Qian, and X. Wu, "Multi-modal multi-view bayesian semantic embedding for community question answering," *Neurocomputing*, 2018.

Click here to access/download
Supporting Information
plosOneFeeConcession.pdf

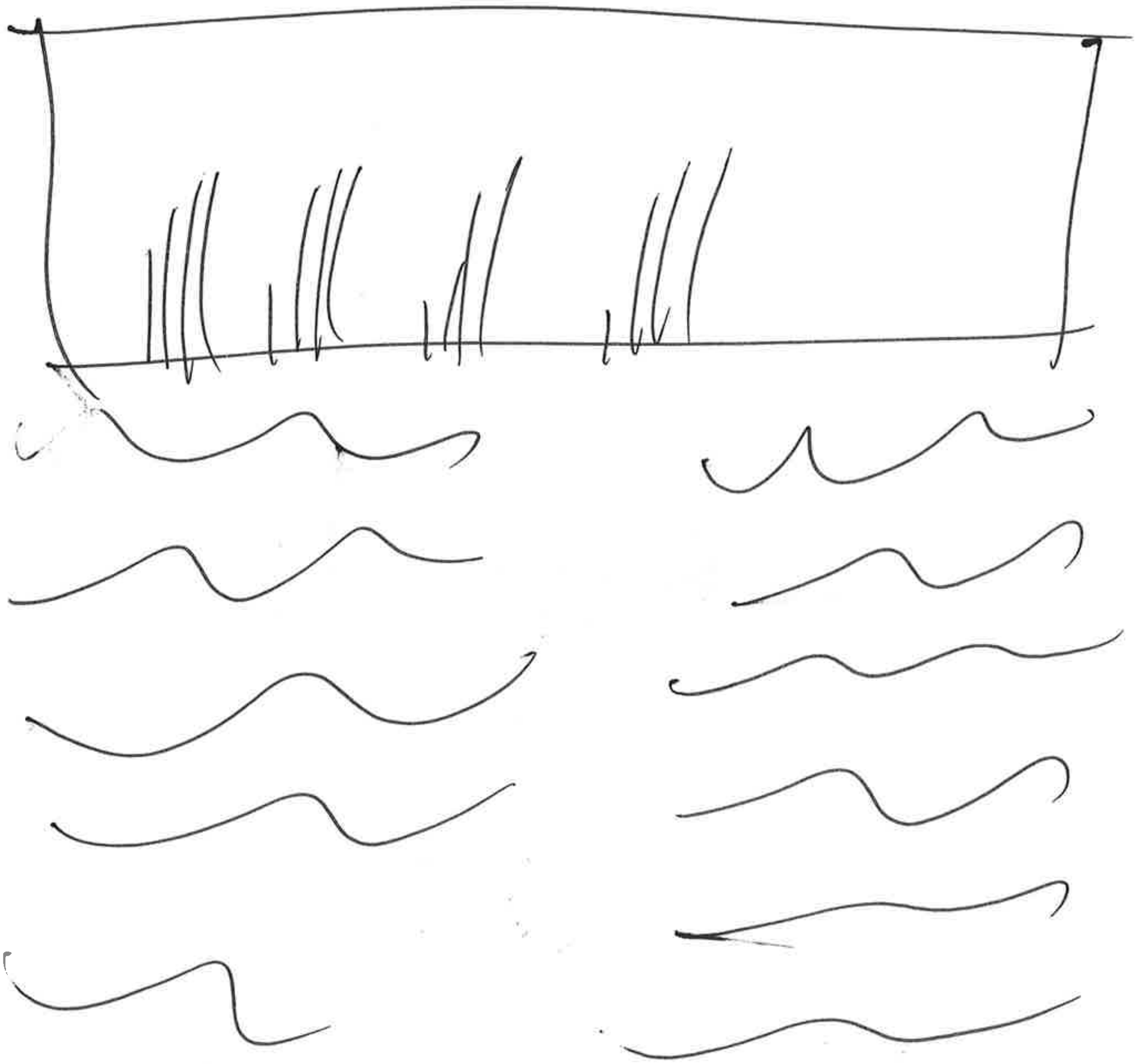

Supplement: Attachment — Submitted filename: 0658_001.pdf [file pone.0264481.s001.pdf]
