# Supplementary material for: Hierarchical lifelong topic modeling using rules extracted from network communities
Source: PLoS One. 2022 Mar 3;17(3):e0264481. doi: 10.1371/journal.pone.0264481 (PMC8893656; doi:10.1371/journal.pone.0264481)
Supplement: Attachment — Submitted filename: Response NHLTMv4.doc [file pone.0264481.s002.doc]

**Comments by the Area Editor**

Thank you for submitting your manuscript to PLOS ONE. After careful consideration, we feel that it has merit but does not fully meet PLOS ONE’s publication criteria as it currently stands. Therefore, we invite you to submit a revised version of the manuscript that addresses the points raised during the review process.

**Response:**

We are very thankful to the reviewers for the detailed comments and suggestions. It really helped us in improving and polishing the manuscript. In the revised version, we have corrected the issues highlighted by the reviewers. In particular, we removed the extra details related to background knowledge of network analysis community and improved the presentation of results for highlighting the strengths and weaknesses in comparison to existing approaches. The lifelong learning aspect of the model is further confirmed by adding additional experimental results on other datasets. Besides this, we have included other useful papers suggested by the reviewers. Below are the some of the newly added papers,

[10] M. J. Paul and M. Dredze, “Discovering health topics in social media using topic models," *PloS one*, vol. 9, no. 8, p. e103408, 2014.

[23] R. Cohen, I. Aviram, M. Elhadad, and N. Elhadad, “Redundancyaware topic modeling for patient record notes," *PloS one*, vol. 9, no. 2, p. e87555, 2014.

[41] J. He, Z. Hu, T. Berg-Kirkpatrick, Y. Huang, and E. P. Xing, “Efficient correlated topic modeling with topic embedding," in Proceedings of the 23rd ACM SIGKDD International Conference on Knowledge Discovery and Data Mining, pp. 225-233, 2017.

[42] Y. Meng, Y. Zhang, J. Huang, Y. Zhang, C. Zhang, and J. Han, \Hierarchical topic mining via joint spherical tree and text embedding,"in Proceedings of the 26th ACM SIGKDD International Conference on Knowledge Discovery & Data Mining, pp. 1908-1917, 2020.

[43] A. B. Dieng, F. J. Ruiz, and D. M. Blei, “Topic modeling in embedding spaces," Transactions of the Association for Computational Linguistics, vol. 8, pp. 439-453, 2020.

[48] Y. Xu, J. Yin, J. Huang, and Y. Yin, \Hierarchical topic modeling with automatic knowledge mining," Expert Systems with Applications, vol. 103, pp. 106-117, 2018.

In the rest of this file, we provide detailed responses to each of the points raised by the reviewers.

**Reviewer # 1 Comments**

**General Comment:**

This paper presents a hierarchical lifelong topic model called NHLTM. This new model combines lifelong learning, with network community detection and topic modelling. Overall I found that the manuscript was well organized and the contributions were clear.
**Response:**

We are thankful to the reviewer for taking time to review the paper and liking it. We tried to incorporate all the concerns of the reviewer and we hope that the revisions in the paper will meet the expectations of the reviewer.

**Comment 1:**

There were several non-native grammar errors. I probably did not catch them all.
**Response:**

We have carefully proof read the paper a few times keeping in view the following points.

1. Grammar and spelling issues.
2. Shorten the long statements.
3. Removed the statement that may cause confusion
4. Remove redundant statements

**Comment 2:**

Section 1 and section 2 are otherwise well organized with only minor typographical and grammatical errors. I did upload my notes to this review. Please look at my ink-notes for detailed comments.
**Response:**

Thanks for appreciating it. We have carefully corrected the typographical and grammatical mistakes pointed out in the attached document. The sentences identified to be rephrased are also written with more clarity and conciseness. Some of the sentences corrected are,

Page 1, Abstract

*Hierarchical topic modeling extends topic modeling by extracting topics and organizing them into a hierarchical structure.*

Page 2, First paragraph

*In this paper we examine a new type of topic model called hierarchical lifelong topic models.*

Page 2, Last paragraph

*On one hand, the rule extraction approaches in LTM models facilitate production of compact and coherent topics but are not well suited to arrange the topics into a hierarchy [8, 11, 12, 13].*

Page 2, Last Paragraph

*Moreover, such support is required each time when new information is processed in the form of new dataset.*

Page 3, 2^nd^ paragraph

*Experimental results on datasets, Chen2014 electronics having 50 categories, non-electronic having 50 categories and R21578 Reuters having 90 categories suggest topic hierarchies with improved topics interpretability [16, 17].*

Page 6, 2^nd^ paragraph

*Hierarchies are highly important in displaying the various components of a system in a tree like structure that has generic concepts at higher levels while specific concepts at lower levels [33].*

Page 11, last paragraph

*NHLTM approach is given in Algorithm 1. It takes the current Dataset Di and a graph G corresponding to the previously processed datasets, i.e., datasets D^1^ to D^i-1^.*

Page 17, 2^nd^ paragraph

*The datasets are preprocessed to retain only meaningful words as verbs, adverbs, adjectives and nouns.*

Page 18, 1^st^ paragraph

*Therefore, aggressive filtering may be needed for filtering out such nodes and edges.*

Page 18, 2^nd^ paragraph

*Filtering out edges with smaller weights and nodes with fewer edges, the graph is refined for efficient analysis. Thus we have a smaller but more densely connected network after the filtering phase.*

Page 21, 1^st^ paragraph

*The value of empirical log-likelihood should preferably increase or stabilize as the model makes better predictions of the unseen data.*

**Comment 3:**

Will the hierarchical structure of a dataset scale logically?

**Response:**

This is a very interesting indication. Logical scaling cannot be applied at community detection for mining rules since the communities do not have any overlap of words. The topics have common words that can be used for drawing their concept hierarchies. But it needs careful evaluation because words occurring in different topics i.e., having different contexts may also be a sign of polysemy. We have added the following line to the manuscript on page 9 in this regards.

*Different approaches can be used for finding the association among rules for improving the arrangement of topics within a dataset and that of words within a topic.*

**Comment 4:**

The topics that do not contain any rules are directly linked to the root of the hierarchy as outliers. How many?

**Response:**

The automated rule mining mechanism allows LTM models to grow in rules with experience. Therefore, the number of relevant rules to process a dataset depends on the number and diversity of the previously processed datasets. Generally, lifelong machine learning faces the problem of cold start where there are not enough rules in the earlier stages to benefit from. We have added the following lines to the manuscript on page 10,

*The topics that do not contain any rule are directly linked to the root of the hierarchy as an outlier. The number of such topics depends on the number of relevant rules in the knowledge base. They are expected to decrease as the number of rules and their diversity increases with experience.*

**Comment 5:**

Reporting raw likelihood values is not normal and not meaningful. Please do not report -856, etc. as results. Just say that it was improved.
**Response:**

This is a very nice suggestion by the reviewer which also becomes evident to us when we reread the paper. We have modified the statements reporting raw values in the abstract and introduction as they were not meaningful.

**Comment 6:**

In section 4 you talk about treating the text corpora as a graph. There exists the hierarchical document topic model (HDTM) which does something similar here that you could look at.

**Response:**

Thanks for pointing it out. We have studied and discussed the relevant literature in the revised now. The relevant text from the page 6 is included below.

Page 6, 3^rd^ paragraph

*It may be noted that hierarchical document topic modeling is also a closely related area where the topic hierarchies are extracted by representing the textual documents in the form of a graph. There are many useful approaches based on hierarchical document topic modeling where some notable ones may be found in the references [46, 47, 48]. The essential assumption in these approaches is that there is an explicit relationship among which can be used to arrange them into a hierarchical structure [49, 50]. Although this assumption is useful in many cases, however, in our study, we do not make this assumption. We only rely on words and their co-occurrence in order to represent the given textual data in the form of a graph.*

[48] Y. Xu, J. Yin, J. Huang, and Y. Yin, \Hierarchical topic modeling with automatic knowledge mining," Expert Systems with Applications, vol. 103, pp. 106-117, 2018.

[49] B. Shi and T. Weninger, \Scalable models for computing hierarchies in information networks," Knowledge and Information Systems, vol. 49, no. 2, pp. 687-717, 2016.

[50] T. Weninger, Y. Bisk, and J. Han, \Document-topic hierarchies from document graphs," in Proceedings of the 21st ACM international conference on Information and knowledge management, pp. 635-644, 2012.

**Comment 7:**

The input of Alg 1, does it require preprocessing of the whole data each time? The input looks like it does.

**Response:**

Thank you for pointing out this problem. This was a mistake which we corrected in the revised version in the updated Algorithm 1 on Page 13. More specifically, the Algorithm 1 gets the current dataset as an input. It also requires as an input a single graph G corresponding to all the previously processed datasets. Each time a dataset is processed, its data is added to the graph, which in turn helps in mining new rules or updating the existing rules.

**Comment 8:**

In general, Section 4 is too verbose. You do not need to describe each line of Alg 1. Alg 2, Alg 3, and Alg 4 are very straightforward and can just be described in prose. Likewise Eq 5 and Eq 6 are well known functions. Just say normalized Laplacian and Fiedler Vector. You don't need to derive it. Any reader will know what those things are. Eq 7 is called "conductance cut" and is well known too - there is no need to describe these things. I think that Section 4 could be reduced to 3-4 pages total.

**Response:**

We have removed Algorithms 2, 3 and 4 from the manuscript that has reduced Section 4 from 10 to 5 pages. Similarly, we have also reduced the commentary on Algorithm 1. The equations that are well known in the community like normalized Laplacian, Fiedler vector, conductance cut, entropy and Lins approximation are now removed and only referred in the manuscript along with their references.

**Comment 9:**

In section 5, you only perform experiments on a single dataset. This is not enough for conclusive study. There exist enormous datasets for study. You need to perform a much more thorough analysis with many datasets.

**Response:**

We have used two other datasets i.e., chen2014 Amazon Non electronic product reviews with 50 categories and Reuters R21578 NLTK corpus with 90 categories for experiments and have compared their results with the existing approaches. We have modified Table 4 to include the Graph properties for each of the three datasets. Figure 3 is also modified to incorporate results of the two newly added datasets when they were allowed to mine rules from 10% of the data and apply it to the new data. We have also modified the discussion on Table 4 and Figure 3 on pages 16-17 and 19-20 respectively as,

Discussion on Table 4 on page 16 and 17

*In this section, we present detailed experimental results of the proposed NHLTM model for extracting topic hierarchies for a sequence of tasks. The Chen2014 dataset with 50 electronic product categories, chen2014 dataset with 50 non-electronic product categories and Reuters R21578 with 90 categories are considered for this purpose [16, 17]. These datasets have documents in multiple categories assumed to be available in a sequence and are used for lifelong topic modeling in literature. In case of the first two datasets i.e., chen2014 Electronic and chen2014 Non-electronic, there are more than 5000 in each category. Each document is a short product review that is collected from Amazon.com. The third dataset has skewed document representation with categories having documents in thousands, hundreds and below that. Each category is considered a dataset for extracting topic hierarchies and are processed in a sequence. Therefore, it fits more into the context of mining rules continuously as an automatic process. Lifelong topic modeling cannot be applied to a single dataset as it requires multiple tasks to improve with experience.*

*Table 4 shows the details of the graphs for each of the three datasets. Only 10% of the past data is considered for mining rules to reduce computational cost. The datasets are preprocessed to retain only meaningful words as verbs, adverbs, adjectives and nouns. The words as nodes, their edges and corresponding weights are mentioned before and after graph filtering. Aggressive filtering is applied to remove weak nodes and their edges as they cannot be part of compact communities which also results in the average edge weight. Filtering out edges with smaller weights and nodes with fewer edges, the graph is refined for efficient analysis. Thus we have a smaller but more densely connected network after the filtering phase.*

Discussion on Figure 3 on page 19 and 20

*The quality of the topics extracted is evaluated using topic coherence. It is the dominant topic evaluation technique that has the highest relevance with human judgment. A higher value indicates better correlation among the words of a topic. The learning curve of lifelong topic modeling is evaluated based on improvement in the quality of topics as topic coherence. Figure 3 shows the comparison of NHLTM with some of the existing approaches including lifelong topic modeling (LTM) [31], automatic must link cannot link (AMC) [34], automatic must link cannot link with must links only (AMCM) [34], online learning based automatic must link cannot link (OAMC) [12] and the basic latent dirichlet allocation approach. All approaches are provided same experience in the form of 10% of the data to mine rules from. They are then presented with a new category as current data to extract interpretable topics by minimizing the impact of noise in the new categories with the help the mined rules. Top 30 words in a topic are used to calculate the topic coherence of a topic. The NHLTM approach has resulted in consistently improving the topic coherence across all the datasets in comparison to others.*

**Comment 10:**

In Table 5, what is the hexadecimal data supposed to show? Why not just represent things as ints? What is Comm Sr. No. column?

**Response:**

In table 5, we have used hexadecimal values to represent the position of a rule in the knowledge-base. Distance between the hexadecimal values of two rule indicate how much apart they are in the knowledge graph. The values are assigned based on the inter-rule associations. While the rules facilitate in improving the interpretability of the topics, their hexadecimal distances help in arranging the topics into a hierarchical structure. Yes, these values can be integers or binary but we have presented them as hexadecimal values for compactness. We added an explanatory statement on page 17 in this regards which is quoted below as,

*The structural position of each community in the graph of all prior data is derived through subsequent splits and hexadecimal codes are used to represent them in Table 5. The communities with smaller difference of hexadecimal codes indicate a nearer common parent and a strong relationship. The codes can be assigned in different ways such as binary or integers but we use hexadecimal values for The sake of compactness.*

Finally, the comm Sr No are basically the ids that we used for identifying the individual communities. They are now being referred to as ID in the revised manuscript in Table 5 on page 18.

**Comment 11:**

Fig 2 us supposed to have some nodes with a grey background, but my printout does not have anything in grey.

**Response:**

In Fig2, the nodes with grey background have not met their stopping criteria and need to be processed further. We have modified the figure to use a darker shade of grey to make them clearly visible.

**Comment 12:**

In Section 5, where do you test the "lifelong" portion of NHLTM?

**Response:**

The lifelong learning aspect of the NHLTM is highlighted by the quality of rules mined by the proposed approach. Figure 3, gives a comparison among the existing lifelong learning approaches i.e., LTM, AMC-M, AMC and OAMC along with the baseline LDA and the proposed NHLTM. We have named the existing approaches in full and have mentioned their references as well. The following lines are modified on page 19 to further clarify this.

*The learning curve of lifelong topic modeling is evaluated based on improvement in the quality of topics as topic coherence. Figure 3 shows the comparison of NHLTM with some of the existing approaches including lifelong topic modeling (LTM) [31], automatic must link cannot link (AMC) [34], automatic must link cannot link with must links only (AMCM) [34], online learning based automatic must link cannot link (OAMC) [12]* and the basic latent dirichlet allocation approach.

**Comment 13:**

Whats the purpose of Table 6? And on page 23 generally, raw values are just not informative.

**Response:**

Table 6 shows the evaluation scores of the communities extracted using spectral clustering. Based on these evaluation scores, some of the communities are filtered while others are retained and preserved as rules. The following lines are modified in the manuscript on pages 18 and 19.

*Table 6 shows other properties i.e., size, entropy, Lins approximation, WGCI and average degree node across community for the corresponding rules in Table 5. These evaluation scores are used to retain only good quality communities with higher compactness within its nodes while filtering out others.*

**Comment 14:**

Fig 3 has the y-axis flipped. It goes from largest on the bottom to smallest on the top. This is quite abnormal.

**Response:**

Thanks for pointing it out. Yes, the values should be from smallest to the largest on the scale along y-axis. We have corrected it.

**Comment 15:**

Table 7 should be a figure.

**Response:**

We converted Table 7 into Figure 4 and table 7 is now being removed.

**Comment 16:**

Conclusions are not strong. What did we learn in this paper? What are the limitations of NHLTM? What is the future work. Expand this section please.

**Response:**

We have modified the conclusion as,

*Lifelong topic models enables the conventional topic model to learn topics continuously from the knowledge accumulated from the past which is updated regularly based on new information. On the other hand, hierarchical topic modeling extends topic modeling for grouping into a hierarchical structure. This study combines the two and proposed hierarchical lifelong topic models which allows the examination of topics at different levels of granularity and continuously adjust the granularity of the topics as more information is made available. The existing hierarchical topic models lack the lifelong learning mechanism and therefore, cannot generate topic hierarchies from the word associations only. On the other hand, the existing LTM approaches do not attempt to generate topic hierarchies. NHLTM has introduced spectral clustering and preserve the communities as rules along with their associations that contribute towards extracting topic hierarchies. A key issue in hierarchical lifelong topic modeling is to extract rules that will not only preserve the hierarchical information among the rules but will also regularly update them as information evolves. We introduced a network community based hierarchical lifelong topic models or NHLTM to address this issue. Experimental results on the datasets of Chec2014, and Reuters R21578 NLTK corpus indicate improvement of the hierarchical topic structures for maintaining high topic coherence that increases from general topics to specific topics.*

*The proposed approach has opened up doors for further research. More specifically, one may extend the NHLTM approach to include topics with multiple parents and to consider polysemy at the rules level. Moreover, the effect of using embeddings in the rule mining component and its impact on the quality of extracted hierarchy may provide further useful insights.*

Overall I think the technical contribution of this paper is ok, but the experiments are severely lacking. Significant additional work is needed before this paper can be accepted.

**Reviewer # 2 Comments**

**General comments**

While interesting, I believe this paper needs major revisions for improved clarity and a more succinct narrative.
**Response:**

We are thankful to the reviewer for liking the work and taking the time for reviewing the paper and constructive comments.

**Comment 1:**

What do you mean by rules? You should present a clear example and definition very early. In one case, it appears that a rule consists of a correlation of two words, but a subsequent equation seems to contradict this.
**Response:**

A rule generally represents the knowledge that we want to carry from a task for possible use in future. The existing literature in lifelong topic modeling used approaches like PMI, FIM etc. and therefore; mined rules as word pairs. Our proposed NHLTM approach use spectral clustering for mining rules and therefore has rules that may consist of any number of words. An explanatory statement is added to page 10, 1^st^ paragraph which is given below,

*It may be noted that the existing studies generally consider rules in the form of word pairs. We also consider rules in the form of word pairs and In addition to that we also consider rules that may contain any number of words.*

**Comment 2:**

Am I right in thinking you used raw frequency? In "classic" approaches to topic modeling, raw frequency is rarely used. It's more common to see something like positive pointwise mutual information (PPMI). Were alternatives to raw frequency explored? If so, why wasn't this reported?

**Response:**

The raw frequencies in Section 3 are for the sake of demonstration to elaborate on the problem i.e., limitations of the existing rule mining approaches for generating topic hierarchies. These are just used for the sake of simplicity in the section 3 only and are not used in the model.

**Comment 3:**

Moreover, typically, term term matrices have a zero diagonal.
**Response:**

Thanks for pointing this out. We have revised the manuscript and we now only use a dash instead of the values. Indeed, this makes more sense as we are not looking for the relationship of the word with itself. The changes may be noted in updated table 2, on page 8.

**Comment 4:**

I recommend that you avoid using "Extract communities using Algorithm 3" when you can instead reference your favored technique by name.
**Response:**

Thanks for suggesting this change. The same changes were also suggested by reviewer 1. In the revised version we have now removed Algorithms 2, 3 and 4 and we now refer to the respective techniques used by their names.

**Comment 5:**

Your tables and figures all warrant real captions so that they can be digested on their own.
**Response:**

Thank you for suggesting this change. We have re-written the captions of tables and figures to make them more meaningful. For instance, we have changed caption of table 1 from,

*Sample data of documents and words*

To

*Sample dataset with 10 documents containing 9 words. A dash (-) indicates the presence of a word in a document.*

**Comment 6:**

Word embeddings have revolutionized natural language processing. Recent approaches to topic modeling that have appeared in venues such as TACL and various ACL conferences have all involved word embeddings to some degree. Why is no discussion or comparison made here? While this might require a second evaluation on another dataset, I think it's necessary.

**Response:**

Thanks for pointing it out. Word embeddings indeed have revolutionized natural language processing. Words and topic embeddings are used in the literature to improve the quality of words within topics and to evaluate correlation among topics. But it hasn’t been used with the LTM approach yet. We consider this a very useful direction for LTM to learn rules based on word embeddings and may even explore rule embeddings. We will consider it as future improvement of our approach. It is also mentioned in the conclusion on page 24 as,

*Moreover, the effect of using embeddings in the rule mining component and its impact on the quality of extracted hierarchy may provide further useful insights.*

Furthermore, we have also added a discussion on word and topic embeddings in the literature review on page 5 which is given below,

*In recent times, word embeddings has improved various natural language processing approaches and is successfully used with topic models [41, 42]. Large text corpora have issues of long tailed vocabularies that result in losing the context. Using topic models on word embeddings help better grouping of words into topics as topic models also attempt to group words based on their context [43]. Topic embeddings are also used to represent topics as dense vectors and help evaluate the correlation among topics [41]. Embeddings are not introduced in the current LTM approaches and may prove to be very effective in preserving context of words within topics and topics within a dataset.*

[41] J. He, Z. Hu, T. Berg-Kirkpatrick, Y. Huang, and E. P. Xing, “Efficient correlated topic modeling with topic embedding," in Proceedings of the 23rd ACM SIGKDD International Conference on Knowledge Discovery and Data Mining, pp. 225-233, 2017.

[42] Y. Meng, Y. Zhang, J. Huang, Y. Zhang, C. Zhang, and J. Han, \Hierarchical topic mining via joint spherical tree and text embedding,"in Proceedings of the 26th ACM SIGKDD International Conference on
Knowledge Discovery & Data Mining, pp. 1908-1917, 2020.

[43] A. B. Dieng, F. J. Ruiz, and D. M. Blei, “Topic modeling in embedding spaces," *Transactions of the Association for Computational Linguistics*, vol. 8, pp. 439-453, 2020.

Lastly, please be noted that the main aim of this research was to investigate hierarchical lifelong topic modelling where the main challenge was to extract rules effectively having both the aspects of having hierarchical structure and lifelong learning.

**Comment 7:**

Speaking of recent approaches, how do neural topic models (ex. VAE-based) compare to your approach? For example, a comparison to Gupta et al's Neural Topic Modeling with Continual Lifelong Learning (ICML 2020) would be nice.

**Response:**

We provided comparison with the existing LTM approaches only as there are some remaining difficulties in including more approaches for comparisons especially those based on neural topic models. The key difficulty is with regards to the implementation which is sometimes not given or not detailed enough or even in some cases not even correct (when someone other than the inventors of the approach provides an implement). The specific algorithmic description such as parameters used to fine tune the algorithm and the experimental setup under which the results are obtained are also not always clear and complete. In addition to these, the datasets used in various studies are also different. Keeping in view these issues, we select a few more approaches that has minimum level of these issues. Since our proposed approach focuses organizing topics into hierarchies over a sequence of datasets, therefore, we have considered LTM models and the HTM models for comparison which provide a reasonable comparative analysis of the proposed approach.

Comment 8:

How does the approach work with different community detection algorithms? Why did you select this particular one without any comparison?

Response:

Comparative studies of community detection algorithms may reveal a winner when carried out in the context of a specific application. Since there were no prior studies on community detection algorithms in the context of our considered application, we are therefore not in a suitable position to confidently select one among the many. However, the selection of spectral clustering in our research was not random and was based on some of its properties that suit more the application we were considering. For instance, Spectral clustering performs better for dense graphs and has reasonable results in various text processing tasks. The graph of multiple datasets with pruning weak nodes and their edges ensures that the graph is dense, thus making spectral clustering more favorable. Moreover, Spectral clustering algorithms are based on computing eigenvalues that allows considering different levels of information and has dimensionally reduction feature (a typical issue faced in textual processing problems). These two properties/features make spectral clustering a more favorable approach for our study. We included an explanatory statement in this regard in the manuscript on page 14 which is given below,

*It is worth mentioning that that there are many community detection algorithms that can be used for extracting communities however, we used the spectral clustering for this purpose. The edges within a community help in identifying useful and important rules while the edges between communities help in identifying the hierarchical association among the rules. The approach continuously updates the communities based on new information which result in updated hierarchical information among the rules. Spectral clustering performs better for dense graphs and has reasonable results in various text processing tasks. The graph of multiple datasets with pruning weak nodes and their edges ensures that the graph is dense, thus making spectral clustering more favorable. Spectral clustering algorithms are based on computing eigenvalues that allows considering different levels of information and has dimensionally reduction feature which is a typical issue in textual processing tasks. All this makes spectral clustering a more favorable approach for our study.*

**Comment 9:**

How are you evaluating the lifelong aspect of your algorithm? AMC-M, AMC and OAMC. Are the abbreviated forms of these acronyms introduced anywhere?

**Response:**

Figure 4 is used to evaluate the lifelong aspect of the algorithm. It basically provides equal experience to all the LTM approaches in the form of a subset of 40 datasets. These approaches are allowed to learn rules with their specific rule mining approaches from the available experience. They are then presented with a new task of extracting topics from a dataset. It shows that the proposed NHLTM approach produces better quality topics that reflect advantage of the newly introduced spectral clustering for rule mining. In the previous version this was not very clear so now we include a clear statement to highlight the lifelong aspect. The relevant text from page 19 is quoted below.

*The learning curve of lifelong topic modeling is evaluated based on improvement in the quality of topics as topic coherence. Figure 3 shows the comparison of NHLTM with some of the existing approaches including lifelong topic modeling (LTM) [31], automatic must link cannot link (AMC) [34], automatic must link cannot link with must links only (AMCM) [34], online learning based automatic must link cannot link (OAMC) [12] and the basic latent dirichlet allocation approach. All approaches are provided same experience in the form of 10% of the data to mine rules from. They are then presented with a new category as current data to extract interpretable topics by minimizing the impact of noise in the new categories with the help the mined rules. Top 30 words in a topic are used to calculate the topic coherence of a topic. The NHLTM approach has resulted in consistently improving the topic coherence across all the datasets in comparison to others.*

And on page 21

*Topic coherence of the proposed NHLTM approach shows higher improvement in down the hierarchy from level one to level six, in comparison to HLDA approach. The topics at higher levels represent generic concepts and therefore, attain lower topic coherence. The lower levels have specific concepts with compact representation. The given results indicate compactness of topics produced by NHLTM at all levels of the hierarchy. The value of empirical log-likelihood should preferably increase or stabilize as the model makes better predictions of the unseen data. The empirical log-likelihood values produced by NHLTM model are maintained lower than HLDA and are also depicted in the Figure 4.*

**Comment 10:**

Table 5: this should be reformatted to make it more obvious which communities are "close".

**Response:**

Table 5 has been reformatted. Indeed, the reformatting makes it clearer and more interpretable. Please be noted that these are some sample communities that have been extracted and not all the communities. These are just shown to have some intuition regarding the extracted communities.

Comment 11:

Please make your code available for reproducibility and future comparisons. Doing so is now standard practice. Ideally, I would like to see how you compared the different models.

Response:

All the code is now being made available online which can be accessed from the link

[*https://drive.google.com/drive/folders/1Ts6S62jpJ7gHlH0JwBjpL6s8ws0QrnaY?usp=sharing*](https://drive.google.com/drive/folders/1Ts6S62jpJ7gHlH0JwBjpL6s8ws0QrnaY?usp=sharing)

**Comment 12:**

Though not a serious problem, there are numerous typographical errors (repeated words, inconsistent formatting, agreement errors, missing determiners, etc.).

**Response:**

We have carefully proof read the paper a few times and have corrected the grammatical and typographical errors.
