# Supplementary material for: Hierarchical lifelong topic modeling using rules extracted from network communities
Source: PLoS One. 2022 Mar 3;17(3):e0264481. doi: 10.1371/journal.pone.0264481 (PMC8893656; doi:10.1371/journal.pone.0264481)
Supplement: Attachment — Submitted filename: Response to Reviewers.doc [file pone.0264481.s003.doc]

**Comments by Reviewer 1**

The author has addressed all of my comments. The new parts of the paper need a thorough proofread - there are minor grammatical issues, but nothing major.

**Response:**

We are thankful to the reviewer for accepting the modifications we have made to the manuscript in response to the comments provided. We have fixed the grammatical issues as highlighted by the reviewer and have proof read the document multiple times.

Below are some of the updates that we made with regards to the grammar in this version of the manuscript.

**Some examples of correct usage of the article.**

‘….50 electronic product categories, Chen 2014 dataset …’ changed to

‘….50 electronic product categories, **the** Chen 2014 dataset …’

‘Filtering out edges with smaller weights and nodes …’ changed to

‘**The** filtering out edges with smaller weights and nodes …’

‘A higher value indicates better correlation among the words of a topic.’ changed to

‘A higher value indicates **a** better correlation among the words of a topic. ’

**‘**All approaches are provided same experience in the … **’** changed to

‘All approaches are provided **the** same experience in the …’

‘Top 30 words in a topic are used to calculate the topic coherence of a topic.’ changed to

‘**The** top 30 words in a topic are used to calculate the topic coherence of a topic.’

‘The communities with smaller difference … ‘ changed to

‘The communities with **a** smaller difference …’

‘ … in comparison to HLDA approach.’ Changed to

‘ … in comparison to **the** HLDA approach.

‘ … The given results indicate compactness …’ changed to

‘ … The given results indicate **the** compactness …’

**Some Examples of adding comma at the right place for better readability**

‘In this paper we examine a new type of topic model …’ changed to

‘In this paper, we examine a new type of topic model …’

‘… meaningful words as verbs, adverbs, adjectives and nouns.’ changed to

‘… meaningful words as verbs, adverbs, **adjectives,** and nouns.’

‘… case of the first two datasets i.e., Chen 2014 Electronic …’ changed to

‘…case of the first two **datasets,** i.e., Chen 2014 Electronic …’

‘… having documents in thousands, hundreds and below that.’ changed to

‘… having documents in thousands, **hundreds,** and below that.’

‘…is derived through subsequent splits and …’ changed to

‘…is derived through subsequent **splits,** and …’

‘Table 6 shows other properties i.e., size, entropy …’ changed to

‘Table 6 shows other **properties,** i.e., size, entropy …’

‘ … generic concepts and therefore, attain …’ changed to

‘ … generic concepts and **therefore** attain …’

**Some Examples of preposition correction**

**‘…** has the highest relevance with human judgment.’ changed to

‘… has the highest relevance **to** human judgment.’

‘… impact of noise in the new categories with the help the mined rules.’ changed to

‘…impact of noise in the new categories with the help **of** the mined rules’

‘ … All approaches are provided same experience in the …’ changed to

‘ … All approaches are provided **with** same experience in the …’

**Some examples of correct singular/plural usage**

‘… are directly linked to the root of the hierarchy as an outlier’ changed to

‘…topics link to the root of the hierarchy as **outliers**’

‘…extracting topic hierarchies and is processed …’ changed to

‘…extracting topic hierarchies and **are** processed …’

‘These type of classes are frequently encountered in …’ changed to

‘These **types** of classes are frequently encountered in …’

**Some examples of correct form of verb**

‘Lifelong topic models enables the conventional …’ changed to

‘Lifelong topic models **enable** the conventional …’

‘This study combines the two and proposed a hierarchical …’ changed to

‘This study combines the two and **proposes** a hierarchical …

‘… word embeddings has improved various natural … ’ changed to

‘…word embeddings **have** improved various natural …’

‘… topic models on word embeddings help better grouping… ’ changed to

‘… topic models on word embeddings **helps** better grouping…’

‘… the communities based on new information which result in updated … ’ changed to

‘…the communities based on new information which **results** in updated …’

‘… computing eigenvalues that allows considering …’ changed to

‘…computing eigenvalues that **allow** considering …’

‘ … of information and has dimensionally reduction …’ changed to

‘ … of information and **have** dimensionally reduction …’

**Some examples of improving the structure of sentence for easy to follow understanding.**

‘Moreover, such support is required each time when new information is processed in the form of new dataset.’ changed to

**‘Moreover, each time new information is available as a dataset, the same support is required.’**

‘Hierarchies are highly important in displaying the various components of a system in a tree like structure that has generic concepts at higher levels while specific concepts at lower levels [33].’ changed to

**‘Hierarchies are crucial in displaying the various components of a system in a tree-like structure. It has generic concepts at higher levels, while specific concepts are at lower levels [33].’**

‘Filtering out edges with smaller weights and nodes with fewer edges, the graph is refined for efficient analysis. Thus we have a smaller but more densely connected network after the filtering phase.’ changed to

‘**The filtering mechanism refines the graph for efficient analysis. It drops the nodes and edges that have a low degree and edge weight, respectively. Thus we have a smaller but more densely connected network after the filtering phase.’**

‘In this section, we present detailed experimental results of the proposed NHLTM model for extracting topic hierarchies for a sequence of tasks.’ changed to

**‘In this section, we present detailed experimental results of the proposed NHLTM model for a sequence of tasks.’**

‘Aggressive filtering is applied to remove weak nodes and their edges as they cannot be part of compact communities which also results in the average edge weight.’ changed to

‘**Through aggressive filtering, the weak nodes and edges are removed as they are least likely to be part of compact communities. It results in increasing the average node degree and edge weight.’**

At the end, we would like to pay thanks again to the editor in chief, associate editor and the reviewers. Indeed, their constructive comments helped to improve the overall quality of the manuscript.
