# Supplementary material for: Hierarchical lifelong topic modeling using rules extracted from network communities
Source: PLoS One. 2022 Mar 3;17(3):e0264481. doi: 10.1371/journal.pone.0264481 (PMC8893656; doi:10.1371/journal.pone.0264481)
Supplement: Attachment — Submitted filename: Round3 Response to Reviewers.doc [file pone.0264481.s004.doc]

**Comments by Reviewer 1**

Grammatical changes appear to be satisfactory. I have no further comments to make regarding the revision of this paper.

**Response:**

We are thankful to the reviewer for his valuable time and feedback. The suggestions were very helpful and contributed in improving the overall quality of our manuscript.

**Comments by Reviewer 3**

This paper proposes an interesting approach for topic modeling. More specifically, the authors put together concepts of NLP and network science to develop a topic modeling approach. Since the author already answered the other reviewers, I have only some points concerning the text.

**Response:**

We are thankful to the reviewer for taking time to review the paper and liking it. We tried to incorporate all the concerns of the reviewer and we hope that the revisions in the paper will meet the expectations of the reviewer.

**Comment 1:**

Since the method involves techniques from different areas, I believe it would be better to explain in more detail some basic concepts. For example, how to calculate and interpret "topic coherence" and "Von Neumann entropy". Note that these concepts are simple, but researchers only from one of the areas involved in this work would not know these concepts.

**Response:**

Thank you for highlighting this issue. This research articles combines multiple research areas and therefore readers from other backgrounds may not be aware of the approaches used for network analysis and topics evaluation. We have provided more detail on them and have included their equations in the manuscript to help assist readers. The original sources are also cited for further details. The following text is being added to the paper;

*“The WGCI of a community comm i can be calculated as,*

$\text{WGCI}\left( \text{comm}_{i} \right)=\sum_{\text{w,w'}} \frac{\text{weight}\left( e_{\text{w,w'}} \right)}{\sqrt{\text{deg}\left( w \right)*\text{deg}\left( \text{w'} \right)}}$	*(3)*

*Where* $e_{\text{w,w'}}$*is the edge between the words* $w$ *and* $\text{w'}$ *belonging to the community* $\text{comm}_{i}$*. In addition to WGCI, we also consider the measure of Von Neumann entropy for evaluating communities [66] as,*

*(4)*

*where N is the total number of nodes in the community and* ${}_{n}\lambda$ *represents their eigenvalues of the normalized adjacency matrix corresponding to the second lowest value. By putting suitable thresholds on the two measures, we selected communities that have considerable associations among its nodes. The threshold values are experimentally chosen to keep only those communities that have higher intra-community associations [67].”*

and page 19 as;

*“The quality of the topics extracted is evaluated using topic coherence. It presents the association or correlation among the words of a topic [71].Thus, the words of a topic that have high contextual correlation results in a higher topic coherence score for the topic. It is the dominant topic evaluation technique that has the highest relevance to human judgment. The topic coherence for a topic k can be calculated as,*

$\text{TC}_{k}=\sum_{\text{w,w'ϵT}_{k}} \text{log}\left( P\left( w|\text{w'} \right) \right)$	*(10)*

*where* $T_{k}$ *represents the k^th^ topic in the given corpus while* $w$ *and* $\text{w'}$ *are two distinct words belonging to the topic* $T_{k}$*. It is the double sum of log of conditional probabilities across all the words in the topic. The top 30 words with highest probability in a topic are used to calculate topic coherence as in literature [71]. The topic coherence of a dataset is determined by averaging the topic coherence over all the topics in a given dataset.”*

**Comment 2:**

On page 12, the authors describe that a co-occurrence graph is used. However, it would help the reader to describe more characteristics of this graph. More specifically, if the edges are directed and/or weighted and how it is determined.

**Response:**

Thank you for pointing this out. We have added more details about the characteristics of network that is maintained across all datasets to identify clusters or community structures from within the network. The following lines are added to the section ‘Representing the data as graph’ on page 12,

*“The algorithm 1 constructs a graph G from the textual data by considering words as nodes and their co-occurrence as edges. In particular, the textual data corresponding to datasets D_1_ to D_i−1_ are used for this purpose. It is an undirected graph as the edges represent the words co-occurrence that can be interpreted both ways. The edges are weighted highlighting the co-occurrence frequency of the two words forming an edge. Considering the power law distribution in the graph where few words have very high occurrence frequency as compared to others and therefore, are more frequent in stable co-occurrence associations. Such graphs can be categorized as scale-free networks that require the weaker filtering the weaker associations. Network based representation has many useful applications in text analytics including keywords extraction, document categorization and knowledge graphs [63]. In this paper, the network of words across all datasets represent a hierarchical knowledge graph that assists topic modeling improving topic coherence and generating hierarchical structure of topics within a dataset.”*

**Comment 3:**

Topic modeling based on communities of complex networks have been studied in: Silva FN, Amancio DR, Bardosova M, Costa LD, Oliveira Jr ON. Using network science and text analytics to produce surveys in a scientific topic. Journal of Informetrics. 2016 May 1;10(2):487-502.
Furthermore, a multi-scale extension of this method was proposed in:
Ceribeli C, de Arruda HF, da Fontoura Costa L. How coupled are capillary electrophoresis and mass spectrometry?. Scientometrics. 2021 May;126(5):3841-51.
Considering that both papers are significantly related to the paper, I think these references could be considered.

**Response:**

Both resources are indeed relevant and further strengthens of the idea of using network analysis for finding structural associations in textual data. The papers are now cited and discussed in the revised manuscript. In the first paper a similar approach is used to determine clusters of articles through community detection in citation network. In our proposed approach we find word clusters from the co-occurrence network. Furthermore, the word clusters help as a source of external knowledge for assisting future hierarchical topic modeling tasks. The suggested paper has other network parameters and evaluation metrics that can also be explored with the proposed approach in future. The second paper also provide network clustering approaches and evaluating their overlap and inter-cluster associations. Thank you suggesting these resources.

**Comment 4:**

In step 5 of section 4.2, it is written: "The α as the smoothing factor." I think that it should be: "α is the smoothing factor." Furthermore, in "Moreover, the β is used as a smoothing factor", I think there is no "the".

Response:

The suggested corrections are made to the manuscript in first paragraph on page 16 as;

α is the smoothing factor.

And

Moreover, β is used as a smoothing factor.

Thank you, very much.
